# Three-dimensional Patterning Super-Black Silica-Based Nanocomposite Aerogels

**DOI:** 10.1007/s40820-025-01870-6

**Published:** 2025-08-20

**Authors:** Zhiyang Zhao, Romain Civioc, Wei Liu, Peiying Hu, Mengmeng Li, Fuhao Xu, Robin Pauer, Jiabei Luo, Samuel Brunner, Paweł P. Ziemiański, Ilia Sadykov, Sandra Galmarini, Yong Kong, Xiaodong Shen, Wim J. Malfait, Shanyu Zhao

**Affiliations:** 1 Laboratory for Building Energy Materials and Components, Swiss Federal Laboratories for Materials Science and Technology, 8600 Empa, Dübendorf Switzerland; 2 College of Materials Science and Engineering, Nanjing Tech University, Nanjing, 210009 People’s Republic of China; 3 Electron Microscopy Center, Swiss Federal Laboratories for Materials Science and Technology, 8600 Empa, Dübendorf Switzerland; 4 Institute of Environmental Engineering, ETH Zurich, Stefano-Franscini-Platz 3, 8093 Zurich, Switzerland

**Keywords:** Super-black, Nanocomposites, Aerogel, 3D printing, Photothermal conversion

## Abstract

Aerogels are ultra-lightweight, porous materials defined by a complex network of interconnected pores and nanostructures, which effectively suppress heat transfer, making them exceptional for thermal insulation. Furthermore, their porous architecture can trap and scatter light via multiple internal reflections, extending the optical path within the material. When combined with suitable light-absorbing materials, this feature significantly enhances light absorption (darkness). To validate this concept, mesoporous silica aerogel particles were incorporated into a resorcinol–formaldehyde (RF) sol, and the silica-to-RF ratio was optimized to achieve uniform carbon compound coatings on the silica pore walls. Notably, increasing silica loading raised the sol viscosity, enabling formulations ideal for direct ink writing processes with excellent shape fidelity for super-black topographical designs. The printed silica–RF green bodies exhibited remarkable mechanical strength and ultra-low thermal conductivity (15.8 mW m^–1^ K^–1^) prior to pyrolysis. Following pyrolysis, the composites maintained structural integrity and printed microcellular geometries while achieving super-black coloration (abs. 99.56% in the 280–2500 nm range) and high photothermal conversion efficiency (94.2%). Additionally, these silica–carbon aerogel microcellulars demonstrated stable electrical conductivity and low electrochemical impedance. The synergistic combination of 3D printability and super-black photothermal features makes these composites highly versatile for multifunctional applications, including on-demand thermal management, and efficient solar-driven water production.

**Supplementary Information:**

The online version contains supplementary material available at 10.1007/s40820-025-01870-6.

## Highlights

- The 3D printed aerogel has an ultra-low thermal conductivity (15.8 mW m^–1^ K^–1^), make it an ideal insulation material in extreme environment (The surface temperature of a 1 cm thickness green body maintained at ≈60 °C after being placed at 300 °C for 30 min).
- The super-black silica-carbon aerogel exhibits surprising light absorption feature (as high as 99.56%), and shows rapid evaporation rate (2.25 kg m^-2^ h^-1^) and excellent energy conversion efficiency (94.2%).
- The combination of super-black and super-insulation features, offering immense potential for multifunctional, high-performance applications across thermal and optical domains.

**Supplementary Information:**

The online version contains supplementary material available at 10.1007/s40820-025-01870-6.

## Graphical abstract

**Figure:**
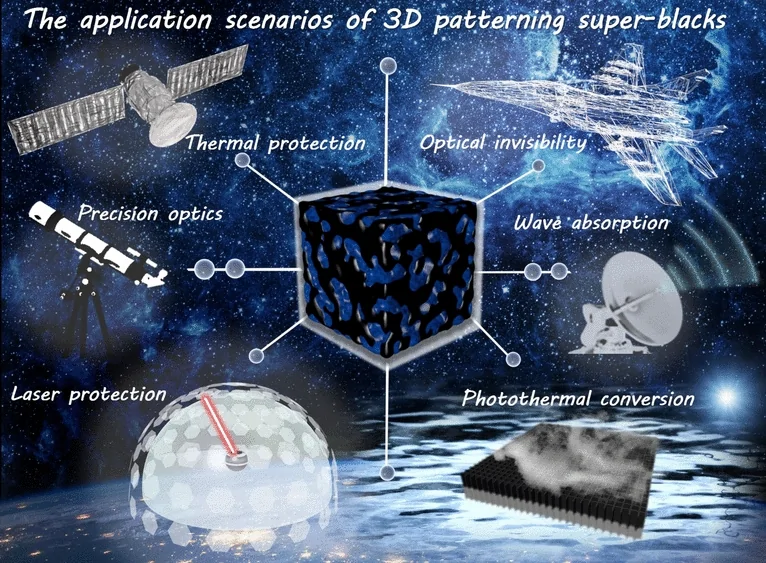

## Introduction

"Super-black" refers to materials or phenomena capable of absorbing over 99% of light, creating the illusion of a "black hole" against its background due to their extreme darkness [1]. The concept of super-black has roots in early scientific discoveries. In 1878, astronomer Samuel Pierpont Langley applied carbon black derived from gas lamp soot to platinum strips for measuring solar radiation [2]. This innovation laid the foundation for the development of super-black materials across various applications, including hydrogen electrodes, solar energy harvesting, and catalytic surfaces [3]. Among artificial materials, aerogels have shown great promise for super-black applications [4]. Aerogels are ultra-lightweight, porous materials characterized by an intricate network of interconnected pores and nanostructures [5]. The intrinsic properties of aerogels—particularly their ultra-high porosity, large specific surface area, and tunable nanoscale network—are pivotal for enabling exceptional functionalities such as efficient mass transport, interfacial interactions, and tailored thermal properties [6]. These features prolong heat transfer pathways, significantly reducing solid thermal conduction, e.g., silica aerogel holds the world record for the lowest thermal conductivity (around 13 mW m^–1^ K^–1^ under ambient conditions) [7]. Moreover, the same porous architecture can trap and scatter light, enabling multiple internal reflections that significantly increase the probability of light absorption by extending the optical path within the material [8]. Carbon is an intrinsic light absorbent [9]. However, controlling the pore structure of carbon aerogels, particularly during pyrolysis, remains challenging, limiting their scalability and uniformity for specific super-black applications. Silica itself lacks intrinsic light absorption, however, by integrating light-absorbing compounds (e.g., carbon nanoparticles [10], graphene [11], or metallic nanostructures [12]), silica composite aerogels can also be engineered to achieve ultra-black properties. Besides, incorporating macro- and micro-scale geometric features (e.g., controlled porosity, specific shapes, surface topography, and microlattices) into materials can significantly enhance or even create new functionalities such as electromagnetic wave absorption [13] and photothermal energy conversion [14].

In addition to selecting materials with high absorptivity, optimizing topographical and geometrical structures can effectively minimize reflection and transmission, thereby enhancing absorption efficiency. Nature serves as a source of inspiration designs, with cellular and vertical structures offering practical advantages due to their ability to promote multiple light reflections [15]. For example, researchers have discovered that the black regions on the wings of male Ulysses butterflies (*Papilio ulysses*) feature microscopic pits less than a micrometer in diameter, effectively contributing to their remarkable optical properties [16]. These pits maximize light absorption by exploiting refractive index differences, creating a blackness surpassing conventional materials. Similarly, the feathers of the magnificent riflebird, native to New Guinea, absorb 99.95% of light due to their hierarchical microstructures, which trap and absorb light, creating a stark contrast with adjacent colorful regions [17]. In artificial materials, such geometrical designs can be achieved through freeze-casting [18] and carved molding [19]. However, recent advancements in topographical design strategies highlight 3D printing as a promising alternative. Unlike freeze-casting, 3D printing offers efficient customization, and compared to molding, it produces smaller, more homogeneous features [20].

We recently explored the integration of silica aerogels with resorcinol–melamine–formaldehyde (RMF) resin to create ultra-black composites [21]. Our findings indicate that the silica-to-resin ratio significantly influences light absorption and darkness [22]. Composites with higher silica content (> 70 wt%) displayed darker coloration, attributed to the formation of small carbon domains on a continuous silica skeleton. This structure minimized shrinkage during pyrolysis and enhanced the material's darkness [15]. Furthermore, silica particles in the resin acted as rheological modifiers, increasing solution viscosity and making it suitable for direct ink writing (DIW) 3D printing [23]. Using DIW, we fabricated complex geometries with excellent shape fidelity. After pyrolysis, the silica–carbon composites retained their structural integrity, exhibited minimal shrinkage, and achieved super-black coloration with high photothermal conversion efficiency [24, 25]. These composites demonstrated excellent mechanical properties, ultra-low thermal conductivity (15.8 mW m^–1^ K^–1^), and stable electrochemical performance. DIW-enabled control over microcellular features allowed us to create super-black porous aerogels for solar-powered water production systems. The hydrophilic, hierarchically porous structure of silica-based aerogels may further enhance vapor condensation efficiency by promoting dropwise condensation, though this study focuses exclusively on evaporation performance. These printed absorbents achieved 99.56% light absorption across the 280–2500 nm range, enhancing evaporation efficiency (2.25 kg m^–2^ h^–1^) and achieving a photothermal conversion efficiency of 94.2%. By optimizing the silica-to-resin ratio and leveraging 3D printing, we developed aerogel microcellular with exceptional light absorption, thermal insulation, and structural stability. These advancements position 3D-printed silica–carbon aerogels as transformative materials for the energy, aerospace, and defense industries, offering immense potential for multifunctional, high-performance applications across thermal and optical domains.

## Experimental Section

### Ink Composition and Preparation

#### Preparation of Anhydrous Formaldehyde Solution

A 37 g of paraformaldehyde (reagent grade; Sigma-Aldrich) was dissolved in 100 g of anhydrous ethanol in a round-bottom flask, then condensed and refluxed at 80 °C for 6 h, until the system changed from a suspension to a clear solution. The water-free formaldehyde solution was stored at 4 °C and homogenized by shaking before use.

#### Preparation of P750P20 Precursor Solution

P750P20 [26], is a pre-polymerized form of tetraethyl orthosilicate (TEOS) containing a water-to-TEOS molar ratio of 1.5 and a SiO_2_ content of 20% w/w in 1-pentanol (referred to as P750P20). The detailed synthesis process is as follows: 173 mL of Dynasylan 40 (ethyl silicate with an SiO_2_ content of 40 wt%, Evonik) was mixed with 94.7 mL of 1-pentanol. A 55 μL of HNO_3_ (ACS reagent, 70%; Sigma-Aldrich) was diluted in 94.7 mL of 1-pentanol to form a dilute HNO_3_ solution. The HNO_3_ solution was injected dropwise (1.58 mL min^–1^) through a syringe pump (Labo Tech Systems) with continuous stirring at 300 r min^–1^. Then, 27.24 g of ultrapure water was slowly added dropwise (0.22 mL min^–1^) into the above solution through a syringe pump, with continuous stirring at 300 r min^–1^. The obtained P750P20 solution was stored at 4 °C for 24 h before use.

#### ***Preparation of SiO***_***2***_***–Resorcinol–Formaldehyde (RF) Ink***

The ink was prepared by first mixing resorcinol (R; 99%; Sigma-Aldrich) and anhydrous formaldehyde solution (F, 37%) with 1-pentanol at room temperature of 25 °C. Then, the P750P20 precursor solution was added and mixed at 500 r min^–1^ for 5 min. Silica aerogel particles (SAPs; amorphous; 5–20 μm; ENOVA, Cabot Aerogel) were added to achieve a certain viscosity required for direct ink writing. The blend was first mixed by spatula and then well homogenized in a planetary speed mixer (DAC 150.1 FVZ; FlackTek) at 1500 r min^–1^ for 10 min. The detailed dosage of inks is shown in Table S1.

### Printing Procedure

The SiO_2_–RF inks were loaded into syringe barrels and de-foamed at 3500 r min^–1^ for 1 min to remove air bubbles inside. The inks were mounted to a direct ink writer from EnvisionTEC (Bioplotter) by a luer-lock to a smooth flow tapered conical polypropylene (PP) nozzles whose inner diameters of 410, 640, and 840 μm. The inks were driven pneumatically through the nozzles at 0.6–3.6 bar, with a filament extrusion rate of 10–18 mm s^–1^. Printing paths and STL files were generated by Magics Envisiontec 18.2, sliced, and converted into BPL files (Bioplotter RP software package) to command the x-y-z motion of the printer head.

### Solidification and Drying

**Fig. 1:**
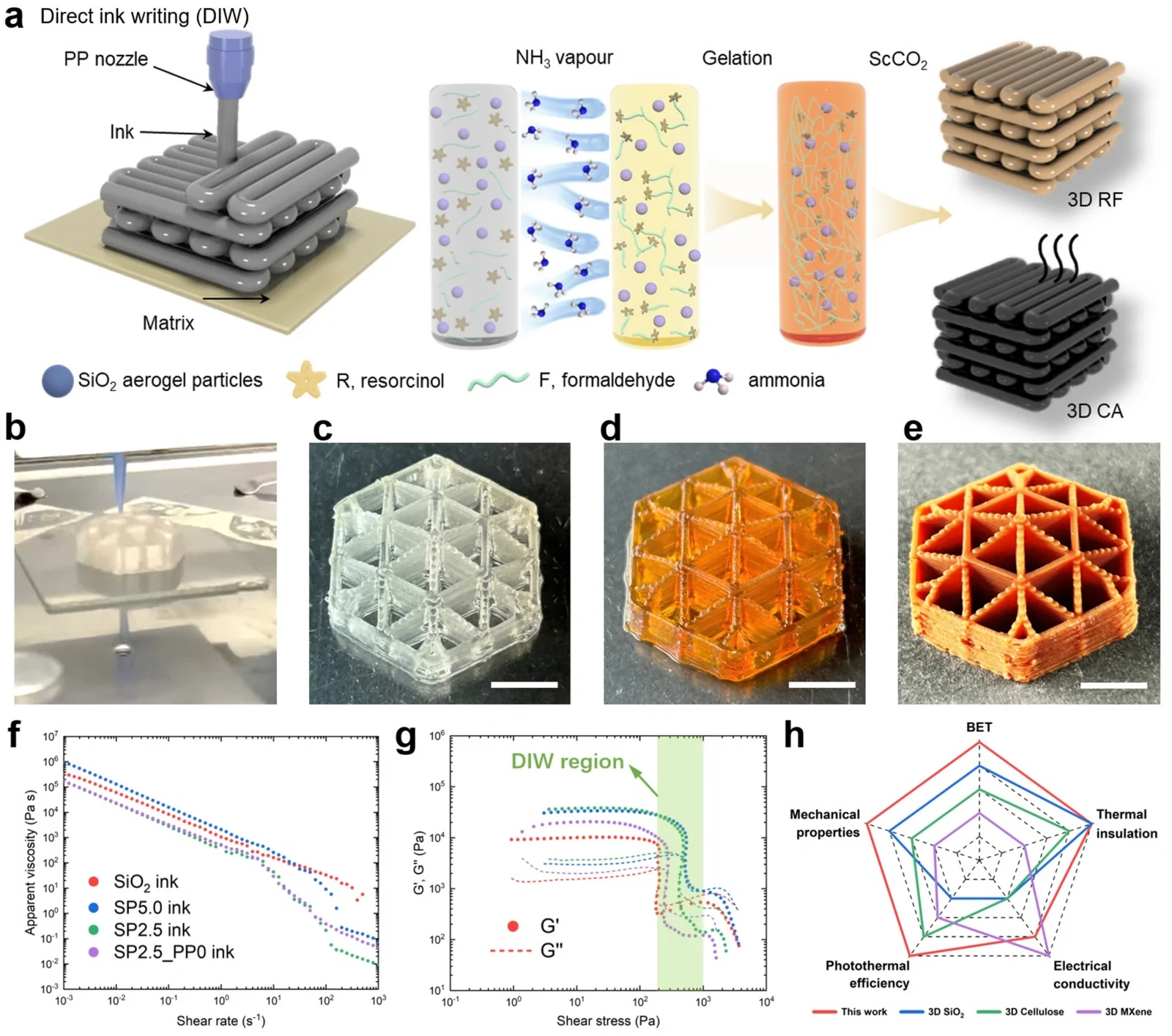
Additive manufacture of SiO_2_–RF composite aerogel by direct ink writing. **a** Scheme for direct ink writing of SiO_2_–RF composite aerogel. The inks are printed smoothly through conic micronozzles. The printed objects are gelled in a NH_3_ vapor-catalyzed system, dried from supercritical CO_2_, and derived carbon–silica aerogels from carbonization. **b** Yurt structure of SiO_2_–RF gel printed from ink SP2.5 (Table S1) through a conical nozzle with an inner diameter of 410 μm, with a flow rate of 12 mm s^−1^. Movie S1 shows a high-speed version of the whole printing process. **c-e** Photographs of the hydrogel from **c** before solidification **d** after ammonia vapor-induced gelation, and **e** after ScCO_2_ drying (the white scale bar is 10 mm). **f** Shear-thinning behavior of different inks. **g** Storage (G′) and loss (G″) modulus versus shear stress for the different inks. **h** The comprehensive performance comparison of the 3D-printed aerogels and other recently reported 3D-printed aerogels, including 3D SiO_2_, 3D cellulose, and 3D MXene aerogels

The solidification of the printed objects was achieved by the ammonia vapor-assisted sol–gel transition of the matrix sol (Fig. 1a). The printed objects were placed in a sealed polystyrene box with a 5.5 M ammonia hydroxide solution that was not in direct contact with the objects. Considering the fact that RF colloids usually formed at a relative high temperature of over 60 °C, and also to avoid cracking and drying of skeleton, the polystyrene box with printed objects and ammonia was placed in a sealed jar containing ethanol solvent and cured in an oven at 65 °C for 72 h. After solidification, color changed from transparent to yellowish, and ethanol (5% isopropanol, Alcosuisse) was used to cover the objects and solvent exchanged in an oven at 65 °C. Finally, the alcoholic objects were placed in a supercritical fluid (SCF) extractor (Separex) for supercritical CO_2_ drying at 120 bar and 50 °C.

### Carbonization

Carbon aerogels (CAs) can be formed through a carbonization step. The green bodies of 3D-printed SiO_2_–RF composite aerogels were heated in a tube furnace under a carbon dioxide atmosphere at 800 °C for 3 h with a heating and cooling rate of 3 °C min^–1^ to achieve the carbonized aerogels.

### Characterization

#### Rheology

The rheological behaviors of the inks at 25 °C were characterized by a rotational rheometer (MCR502, Anton Paar) with a 25-mm diameter steel plate geometry and a controlled gap of 0.5 mm. Apparent viscosities were measured through a steady-state flow method with shear rates varying from 0.001 to 1000 s^−1^. Shear storage and loss moduli were tested as a function of shear stress through an oscillatory method at a fixed frequency of 1 Hz with shear stress changing from 0.001 to 10,000 Pa. All the inks were equilibrated at room temperature of 25 °C for 1 min before testing.

#### Thermal Conductivity

Thermal conductivity tests were determined from square plate specimens of printed monolithic aerogels, in which the sample sizes are about 50 mm × 50 mm and 10 mm in thickness. After post-curing and drying, the aerogels were placed in a custom-built guarded hot-place device (Fig. S1) designed for small samples of low thermal conductivity materials (guarded zone: 50 mm × 50 mm; measuring zone: 25 mm × 25 mm; room temperature of 25 °C) [27]. For matters of comparability, a number of commercially available insulation materials were tested via this device to evaluate their thermal conductivity.

#### Microstructural Characterization

Scanning electron microscopy (SEM) images were recorded on a ZEISS GeminiSEM 460 at an accelerating voltage of 5 kV and a current of 100 pA. The typical working distance was controlled 2–5 mm. Nominally 15 nm of platinum (Pt, measured with a flat piezo detector) was coated to avoid charging, but the effective thickness on the aerogels, with their extreme topography and surface area, will be much lower. Nitrogen sorption analysis was carried out on a TriFlex nitrogen sorption analyzer (Micromeritics) after prior degassing for 8 h at 120 °C and 0.02 mbar. The specific surface areas (*S*_*BET*_, uncertainty ≈50 m^2^ g^–1^) were obtained using the BET method. The pore volume (*V*_*0*_) and average pore diameter (*d*_*0*_) were calculated from the density of the printed aerogels and their *S*_*BET*_ using Eqs. (1) and (2), respectively.

1 $${V}_{0}=\frac{1}{\rho }-\frac{1}{{\rho }_{skeleton}}$$

2 $${d}_{0}=\frac{4{V}_{0}}{{S}_{BET}}$$

where *ρ* is bulk density, *ρ*_*skeleton*_ is skeletal density, and *S*_*BET*_ is specific surface area.

#### FTIR Analysis

ATR-FTIR measurements were performed on a spectrometer from Bruker Switzerland AG with the ATR name Tensor 27 over the wavenumber range from 400 to 4000 cm^–1^.

#### X-Ray Diffraction Analysis

The X-ray diffraction analyses of the printed aerogels and derived carbon aerogels were recorded on a PANalytical X’Pert PRO diffractometer equipped with a Johansson monochromator (Cu Kα1 radiation, λ = 1.5406 Å).

#### Thermal Stability

Thermogravimetry analysis was performed on a thermogravimetric analyzer (TG 209 F1, NETZSCH) in an air atmosphere, with a constant heating rate of 10 °C min^–1^ from room temperature to 900 °C.

#### Contact Angle

The surface wettability of aerogels was evaluated by using a contact angle measurement system OCA (Dataphysics TBU 90E, Germany), combined with a high-speed camera. Water droplets (10 μL) were deposited directly on the surface of the aerogels. Three measurements were performed and averaged.

#### Dynamic Vapor Sorption

Dynamic water vapor adsorption and scanning desorption were measured by an automated vapor flow sorption balance device (VTI-SA + , TA Instruments, USA). Approximately 10–15 mg of the composite aerogels were first dried for 6 h at 120 °C and at a partial water vapor relative humidity (RH) of 0. The samples were then exposed to ascending RH steps of 0%, 5%, 15%, 25%, 35%, 45%, 55%, 65%, and 75% for adsorption and then descending in the same manner for desorption at 50 °C. Equilibrium in each step was defined to be reached at a mass change per time (dm/dt) of less than 0.001% min^–1^ over a 10 min window or a maximal time of 300 min per step. The samples were exposed to a flow rate of 0.2 L min^–1^, with dry air (grade 5.0) as the carrier gas.

#### Mechanical Properties

The mechanical characterization (uniaxial compression) of aerogels was performed on three identical cylinders (diameter, 10 mm; height, 15 mm). The processed aerogel cylinders were polished to even the surfaces for uniaxial compression testing. The specimens were tested on a mechanical testing machine (Z010, Zwick/Roell, Germany) with a 5-kN force transducer (KAP-S, AST Gruppe GmbH, Germany) at a constant rate of 1 mm min^−1^. Stress–strain curves were recorded in compression mode, and elastic moduli were calculated from the linear regime of the curves which typically occurred at 7 ± 3% strain. The samples were compressed up to 80% strain or until the first buckling event with > 50% loss of stress. The reported final compressive strength values correspond to the maximum stress reached during the experiment.

#### Infrared Thermal Imaging and Thermal Management

The infrared images in high-temperature range were recorded using Testo 880–1 (measuring range 0–350 °C). Thermal couple (RS PRO RS-172TK Temperature & Humidity Data Logger) was used to record the temperature. The thermal insulation performance with heat and cold source was evaluated using an infrared camera (TH 3102 MR, NEC-San-ei; measuring range −50–550 °C) equipped with a Stirling-cooled HgCdTe detector, with a temperature sensitivity of 0.08 at 30 °C and an accuracy of ± 0.5 °C. The emission was set to 1. Thermal images were analyzed on a PicWin-IRIS system (version 7.3). The object with sizes about 5.0 cm × 5.0 cm and thickness of 1.0 cm.

#### Light Absorption Test

The light absorbance spectra of the samples were measured using a UV–Vis–NIR spectrophotometer (UV-3600, Shimadzu) equipped with an integrating sphere over the wavelength range of 280–2500 nm. BaSO_4_ was used for baseline and, in the case of reflectance, a Spectralon was used as reference. The spectral absorbance was calculated using the following equation:

3 $$Absorbance \, = \, 1 \, {-} \, \left( {Reflectance \, + \, Transmittance} \right)$$

#### Photothermal Conversion Test

The experiments of photothermal conversion were carried out under room temperature. Xenon lamp light source system (CEL-HXF300-T3, Beijing Zhongjiao Jinyuan Technology Co., Ltd.) was used to simulate the sunlight under 1.0 sun illumination. Solar intensity was calibrated by a solar power meter (SM206-SOLAR). The floating melamine sponge was used both as the water channel and also isolate the carbon aerogel from water surface. The mass change during evaporation was measured by an electronic balance and recorded once every 5 min. The top temperatures of carbon aerogel were measured by infrared thermal imager, and the ambient temperature was measured by a thermometer. The evaporation rate and the energy efficiency (η) for solar vapor conversion thus can be obtained (the detailed steps are listed in Note S1).

#### Electrical Properties

The electrical conductivity of the samples was evaluated by a multimeter (UNI-T UT58E). Set the multimeter to resistance mode at 2 MΩ, then place the probe on the sample surface, and record three measurements at different points for precision. To visualize conductivity, conductive clips are attached to the sample and connected to a circuit with LED bulbs. Changes in the brightness, shifting between light and dark, reflect the conductivity of the printed material. To characterize the impedance and its stability, the electrochemical workstation (Biologic SAS, VSP-300, France) was used with the size of the aerogel electronics at 10 mm × 10 mm × 1 mm.

#### Electromagnetic Wave Absorption Properties

The vector network analyzer (VNA, PNAN5244A, Agilent, USA) was employed for measuring the dielectric constant. The sample and paraffin wax were homogeneously blended at a 2:8, 3:7, 4:6, and 5:5 mass ratio at 70 °C. Coaxial ring specimen, with outer and inner diameters of 7 mm and 3 mm, was made from the resulting mixture. The dielectric constant was evaluated via the transmission line technique across a frequency band of 2.0–18.0 GHz. The acquired dielectric constant was then employed to calculate the attenuation constant (α) and RL_min_. The values of reflection loss (RL), determined according to transmission line theory and computed from the relative complex permittivity and permeability at specific frequencies and matching thicknesses from Eqs. (4) and (5), are employed to assess EWA performance [28].

4 $$RL=20\mathit{lg}\left|\frac{{Z}_{in}-{Z}_{0}}{{Z}_{in}+{Z}_{0}}\right|$$

5 $${Z}_{in}={Z}_{0}\sqrt{\frac{{\mu }_{r}}{{\varepsilon }_{r}}}\mathit{tan}h\left({j}\frac{2\pi fd\sqrt{{\mu }_{r}{\varepsilon }_{r}}}{c}\right)$$

where *Z*_*in*_, *Z*_*0*_, *ε*_*r*_, *μ*_*r*_, *f*, *c*, and *d* represent the input impedance of the absorbent, free space impedance, the complex permittivity, permeability, incident EMW frequency, light speed in a vacuum, and matching thickness, respectively.

In accordance with the free-electron theory and the Debye theory, when there exists the dielectric polarization relaxation, the relationship of *ε′* and *ε″* satisfies the following functional relationship:

6 $$\varepsilon^{\prime } = {\upvarepsilon }_{\infty } + \frac{{\varepsilon_{s} - \varepsilon_{\infty } }}{{1 + \omega^{2} \tau^{2} }}$$

7 $$\varepsilon^{\prime \prime } = \varepsilon_{p}^{\prime \prime } + \varepsilon_{c}^{\prime \prime } = \frac{{\varepsilon_{s} - \varepsilon_{\infty } }}{{1 + \omega^{2} \tau^{2} }}\omega \tau + \frac{\sigma }{{\omega \varepsilon_{o} }}$$

8 $$\left( {\varepsilon^{\prime } - \frac{{\varepsilon_{s} + \varepsilon_{\infty } }}{2}} \right)^{2} + \left( {\varepsilon^{\prime \prime } } \right)^{2} = \left( {\frac{{\varepsilon_{s} - \varepsilon_{\infty } }}{2}} \right)^{2}$$

where *ε*_*∞*_ denotes the relative permittivity at infinite frequency, *ω* presents the angular frequency, *ω* = *2πf*, *τ* represents the relaxation time, *ε*_*s*_ stands for the static permittivity, *ε*_*p*_*″* and *ε*_*c*_*″* express the polarization loss and conductivity loss, *σ* is the electrical conductivity, and* ε*_*0*_ is the permittivity of vacuum. *ε'* primarily pertains to the polarization loss, whereas *ε"* encompasses the combined effects of polarization loss and conductivity loss.

## Results and Discussion

### SiO_2_–RF Ink Behavior and Printing Performance of the Green Bodies

For creating a super-black carbon–silica composite, the silica concentration is critical (Fig. S2). With good control of the silica-to-carbon proportions (exceeding 70 wt% silica concentration), we can achieve deep black color as well as superior insulation (Fig. S2, Table S2). The silica particles added can also serve as a filler phase to improve the rheology in an ink formulation for DIW.

Considering the classic silica and RF chemistries, hydrophobic silica particles are incompatible with the water solvent typically used for RF resin, hydrophilic silica is also not ideal in polar solvents because the surface-covered hydroxyl groups can induce particle aggregation [29]. Therefore, to create a printable ink, an alcoholic solvent mixture of ethanol and 1-pentanol was adapted for the preparation of RF. The fabrication of these alcoholic-based inks for SiO_2_–RF composite aerogels, along with their printing and carbonization processes, is illustrated in Fig. 1a–e. We print SiO_2_–RF composite aerogel objects using DIW of a hydrophobic silica aerogel powder in a 1-pentanol/ethanol-based SiO_2_–RF sol (Fig. 1a) [30]. The low surface tension of 1-pentanol prevents surface cracks and shrinkage during the drying process, especially when printing complex structures (Figs. 1b and S3). The printed objects are gelled in an NH_3_ vapor-catalyzed system, dried using supercritical CO_2_, and then pyrolyzed in a CO_2_ atmosphere.

The freshly printed wet gels are colorless and transparent (Fig. 1c). Objects have been printed with filament and nozzle diameters as small as 100 μm. Smaller diameters are feasible, given the aerogel powder particle size is as small as 10 μm (Fig. S5), but the printing system will need to operate at very high pneumatic pressure. The rheological behavior is complex: strain overshoot at large-amplitude shear, typical for colloidal suspensions [31], and non-Newtonian shear thinning at small-to-medium-amplitude oscillatory shear, typical for suspensions of large particles (Fig. 1f). The silica aerogel particles increase the ink's viscosity, preventing solid–liquid phase separation, and improve homogeneity during the sol–gel transition. With an increase in silica particles, the apparent viscosity also increases, requiring more printing pressure during DIW. There is no significant difference in both the viscosity and storage modulus of the silica composite inks compared to the reported silica inks [23], indicating a printable silica composite ink (Fig. 1g). After ammonia vapor-induced gelation, RF polymerization begins, changing the color to yellowish and translucent (Fig. 1d). After ScCO_2_ drying, the resulting silica composite aerogels are yellow but opaque (Fig. 1e). Following activation and carbonization in a CO_2_ atmosphere, deep black carbon composites are obtained. Thanks to ScCO_2_ drying technology, which minimizes damage, the sol-to-gel shrinkage remains constant. Notably, when silica aerogel particles are added to the inks (*e.g.*, SP2.5 ink listed in Table S1), the derived carbon composite aerogel maintains the printed features and shows a linear shrinkage of 28% (Figs. S6 and S7). Such design and manufacturing of RF–silica aerogel and derived porous carbon composites were confirmed with superior combination over various state-of-the-art 3D printing aerogels (Fig. 1h), including 3D-printed SiO_2_ [23], 3D cellulose [32], and 3D MXene aerogels [33], the detailed characterization and comparison were presented in the following sections.

### Microstructure of the Printed SiO_2_–RF and Derived Carbon–SiO_2_ Composite Aerogels

**Fig. 2:**
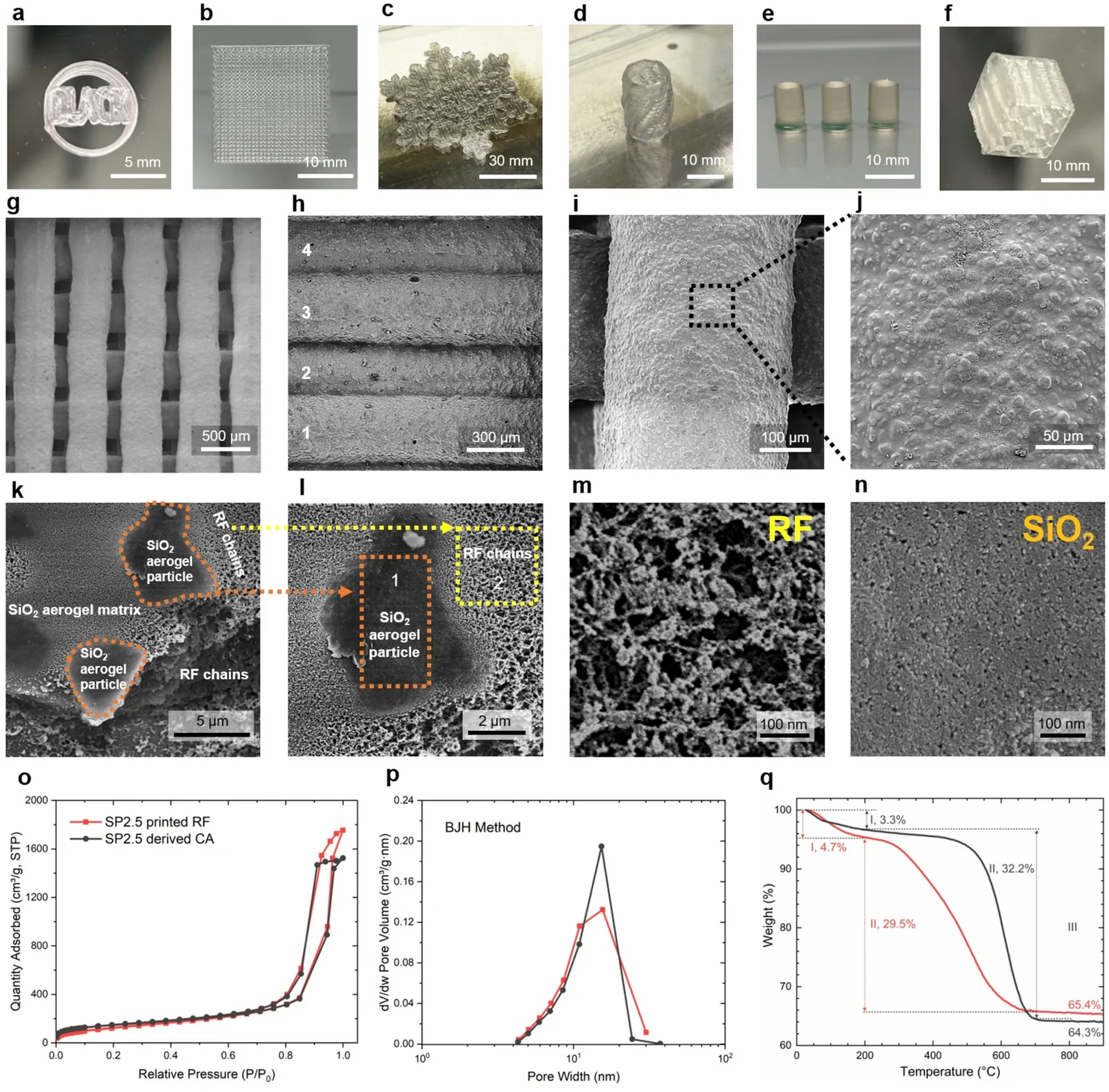
Three-dimensional-printed objects, their microstructure and selected properties. **a-f** Various 3D patterns of **a** character, **b** lattice, **c** snowflake, **d** vase, **e** cylinders, and** f** cube (ink SP2.5, 410-μm PP nozzle). **g** Scanning electron microscopy (SEM) image of a lattice. **h** A multi-layer continuous membrane. **i** Single printed filament. **j** Magnification of the outer surface of single printed filament. **k** Interface between two filaments. **l** Magnification of the orange-cycled region in **g**, showing aerogel particles embedded with RF chains. **m** Magnification of the yellow-boxed region in **l**, highlighting the RF phase. **n** Magnification of the orange-boxed region in **l**, highlighting the SiO_2_ phase. **o** N_2_ sorption isotherms at 77 K. **p** Pore size distribution derived from Barrett–Joyner–Halenda (BJH) analysis. **q** Thermogravimetric analysis, weight change I corresponds to adsorbed water, weight change II to organic groups

The developed inks exhibit a broad DIW printability window, enabling the printing of large and complex geometries. Various aerogel objects with high shape fidelity and precision have been successfully printed (Fig. 2a–f), including character, lattice, snowflake, vase, cylinders, and 3D cubes. The printed filaments retain a circular cross section with a well-defined diameter of 391 ± 15 μm from a 410-μm conic nozzle (Fig. 2g), indicating minimal volume loss after solidification and drying. Additionally, the good rheological behavior of the ink allows filaments to merge into continuous membranes without voids (Fig. 2h). The original SiO_2_ aerogel particles are embedded in, and infused by, a low-density aerogel composite matrix derived from both the RF and silica sols (Fig. 2k, l), resulting in an aerogel composite surface that appears unsmooth (Fig. 2i, j). The final ink formulation imparts consistent rheology and high shape fidelity printing with over 70 wt% silica, which also meets the deep black carbon composite criteria (Fig. S2 and Table S2). The RF resin (Fig. 2m) also shows a continuous coating on the particulate silica aerogels (Fig. 2n), and both exhibit high meso-porosities. SEM images in Fig. S10 depict that even after high-temperature activation and pyrolysis, the high meso-porosity microstructure is well maintained. Transmission electron microscopy (TEM) images in Fig. S10 also show the expected colloidal natural and random aggregation behavior for both silica and carbon network. Meanwhile, no obvious lattice and diffraction fringes exist in TEM images, indicating the amorphous form of SiO_2_ and carbon in the derived aerogel network. The nitrogen sorption isotherms of the printed SiO_2_–RF composite aerogel and derived carbon aerogels are similar. The specific surface areas of the printed aerogels before and after pyrolysis decrease from 556 to 517 m^2^ g^−1^ and the average pore sizes increase from 9.1 to 13.4 nm, respectively (Fig. 2o, p). The printed SiO_2_–RF green body (printed with ink SP2.5) has a relatively low density (0.16 ± 0.02 g cm^−3^), and can easily stand on the flower (Fig. S4). After activation and pyrolysis, the corresponding carbon composite density increases to 0.25 ± 0.03 g cm^−3^, due to the conversion of organic RF to carbon during these processes. Both the printed composite aerogels can be divided into three regions in the TGA curves (the qualitative analysis are listed in Note S2). Compared to the SiO_2_–RF composite aerogel, the derived carbon–SiO_2_ composite aerogel exhibits greatly improved thermal stability at high temperatures (Fig. 2q), with substantial loss of organic groups occurring above 500 °C. The printed samples, including the starting RF composite aerogel and the derived carbon aerogel, have similar breaking elongations (~ 10%) as standard silica aerogels but possess better machinability (Fig. S11a), due to the large proportion (> 70 wt%) rigid silica network. Benefiting from RF polymer reinforcement, both the printed RF composite aerogel and the derived carbon aerogel exhibit higher mechanical strength. The maximum compressive strength is 613 and 1274 kPa, respectively. Carbonization improves the Young’s modulus by a factor of twelve (Fig. S11b, c). Consequently, the printed RF and their derived carbon composite aerogels exhibit enhanced mechanical properties compared to previously reported silica aerogels [34–36], cellulose aerogels [37–39], and biopolymer aerogels (Fig. S11d) [40, 41].

### Thermal Management with SiO_2_–RF Composite Aerogels

**Fig. 3:**
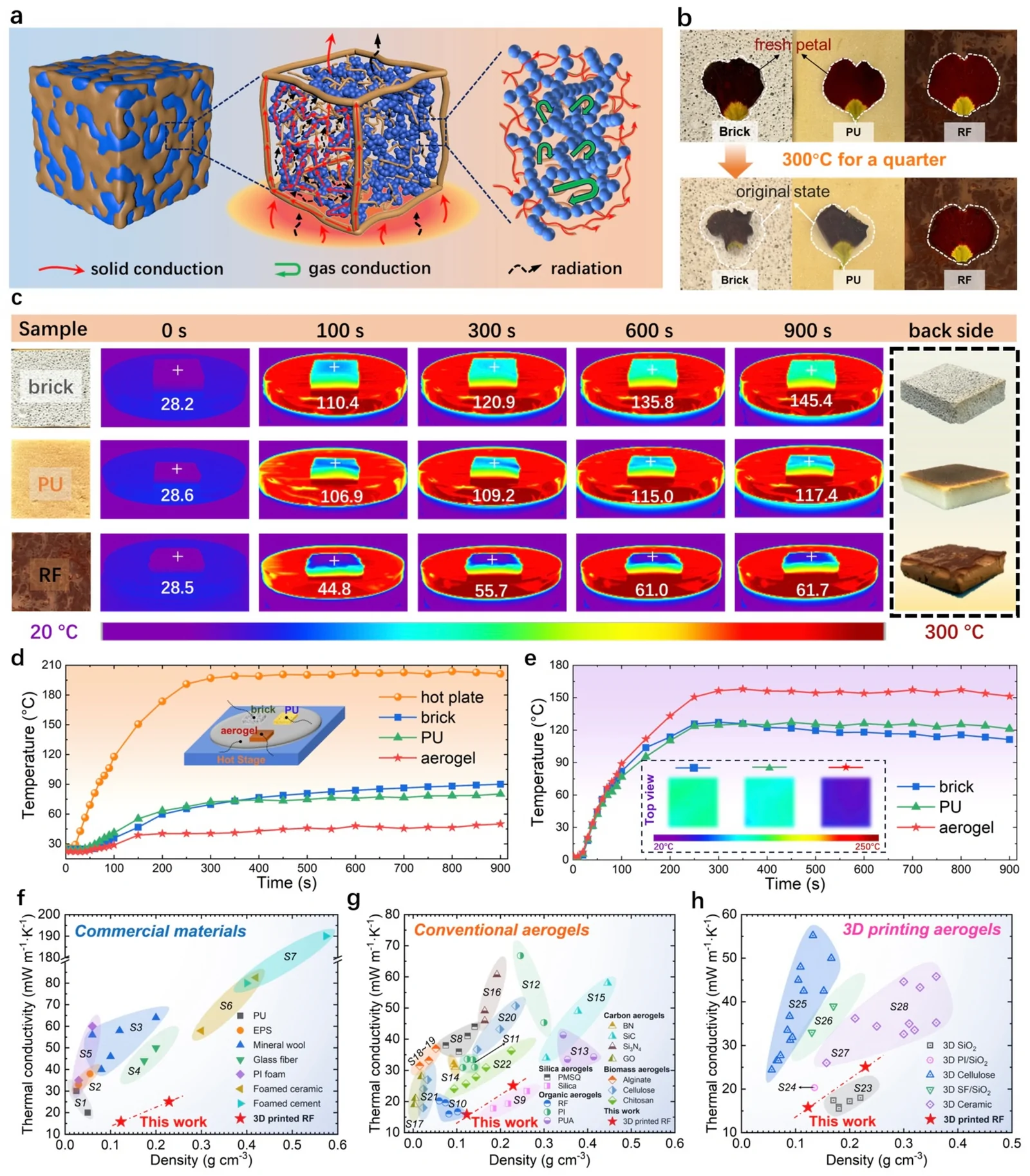
Thermal management of SiO_2_–RF composite aerogel. **a** Thermal insulation mechanism of SiO_2_–RF composite aerogel with dual-network structures. **b** Comparison of thermal insulation performance of SiO_2_–RF composite aerogel, PU foam, and building brick materials. **c** Optical and infrared images of SiO_2_–RF composite aerogel, PU foam, and building brick materials heated at 300 °C for 15 min, and their backside optical images after heating. **d** Temperature–time curves of SiO_2_–RF composite aerogel, PU foam, and building brick materials at a stable temperature of 300 °C (inset: schematic diagram of the hot stage test). **e** Temperature difference–time curves of SiO_2_–RF composite aerogel, PU foam, and cement brick materials at a stable temperature of 300 °C (inset: infrared image of the upper surface of the samples under steady state). **f–h** Comparison of thermal conductivities of 3D-printed SiO_2_–RF composite aerogels with **f** commercial insulation materials, **g** conventional aerogels, and **h** 3D printing aerogels (Table S3)

Before converting the printed green body to a carbon composite, we are also interested in the thermal properties of the SiO_2_–RF composite aerogel, owing to the thermal stability of its silica skeleton. Thermal transfer is commonly divided into three forms (Fig. 3a): conduction (including gas conduction λ _g_ and solid conduction λ _s_), convection (λ _conv_), and radiation (λ _rad_) [42, 43]. The average pore size in RF composite aerogel is 9.1 nm, and we cannot identify many large macroscopic structures from the SEM (Fig. 2k-n). Therefore, λ _conv_ does not occur, and λ _g_ could also be significantly suppressed. Although at room temperature, λ _rad_ can also be ignored, but at high temperatures [44, 45], RF and carbon materials are effective IR opacifier to suppress λ _rad_. For solid thermal conduction [46], due to the low density of the SiO_2_–RF composite aerogel (0.123 g cm^−3^), solid conduction is restricted by blocking phonon conduction pathways. The interfacial thermal resistance (known as Kapitza resistance) [47], originating from phonon scattering between fiber-like RF chains and amorphous SiO_2_ nanoparticles, can further reduce the solid conduction of the composite aerogel. The heat transfer occurs through thermal conduction, thermal convection, and thermal radiation. When the SiO_2_–RF composite aerogel comes into contact with the heating table, the porous structure of the aerogel delays thermal conduction. The large specific area of the aerogel can significantly radiate heat to the surroundings. When thermal convection occurs, abundant air exists in the SiO_2_–RF composite aerogel, which can effectively block the heat transfer and keep the surface layer of aerogel at a low temperature. For carbon composite aerogels, the thermal insulation mechanism involves synergistic effects across multiple scales. At the molecular level, the mesoporous silica framework (pore size≈20 nm) significantly suppresses gaseous conduction by restricting the mean free path of air molecules, consistent with Knudsen diffusion theory. Meanwhile, the nanoscale carbon coating selectively scatters phonons at Si–C interfaces while maintaining electron percolation, effectively decoupling electrical and thermal transport as demonstrated in [48]. Critically, the hierarchical porosity generates strong Mie scattering centers that attenuate infrared radiation (> 90% reduction at 3–5 μm), aligning with the radiative regulation principle reported by [49]. This multi-scale strategy achieves an ultra-low thermal conductivity of 25.1 mW m^−1^ K^−1^. Overall, both the green body aerogel and the converted carbon aerogel exhibit excellent thermal insulation properties, making it well-suited for either high-temperature or harsh environments.

In order to demonstrate the excellent insulation potential, squares of RF composite aerogel, PU foam, and cement brick of the same height (10 mm) were placed on a heating stage at 300 °C (Fig. S12), and fresh petals were placed on top of each material. As depicted in Fig. 3b, the petals on the aerogel exhibited only a slight dehydration and wilting at the boundary after heating for 15 min, whereas those placed on other materials were charred and carbonized. By comparing the petals to their original outlines (the white dotted area in Fig. 3b) and start-and-end states, it is not difficult to indicate the superior thermal insulation performance of the RF composite aerogel at high temperatures. Further, we monitored the dynamic temperature changes shown in Fig. 3c, a temperature gradient distribution from the heating plate to the RF composite aerogel was observed. The RF composite aerogel (10 mm thick) was kept at a low temperature of ≈55.7 °C after being kept on a 300 °C heating plate for 5 min. The temperature slightly increased to 61.0 °C after 10 min and remained unchanged after 15 min. As a comparison, the upper surface temperatures of PU and cement brick samples under steady-state (15 min) conditions are 117.4 and 145.4 °C, respectively. Under long-term high-temperature exposure, the back side of the building brick turns yellow, the PU is burnt to black, only the printed RF composite aerogel remains unchanged, demonstrating the high-temperature stability. In the same experiment, the temperature evolution profile was monitored by thermocouples (Fig. 3d)**,** the SiO_2_–RF composite aerogel had the slowest heating rate and the lowest plateau temperature among the three samples. The specific temperature differences (ΔT) of the three thermal insulation materials at high hot stage temperatures are shown in Fig. 3e, the largest ΔT (150 °C) in the SiO_2_–RF composite aerogel also confirms its good temperature resistance at high service temperatures.

The thermal conductivity of the printed SiO_2_–RF composite aerogel at room temperature is 15.8 mW m^−1^ K^−1^, well below that of standing air (26 mW m^−1^ K^−1^), and comparable to the best superinsulating silica aerogels (15 mW m^−1^ K^−1^). We compared the thermal conductivity of printed RF composite aerogel with different thermal insulators including commercial materials, aerogels through traditional mold casting process, and diverse 3D printing aerogels at room temperature. The detailed values are listed in Table S3. Compared with commercial materials such as polyurethane (PU), expandable polystyrene (EPS), mineral wool, glass fiber, polyimide (PI) foam, foamed ceramic, and cement (Fig. 3f), the printed SiO_2_–RF composite aerogels show a significantly lower value both in density and thermal conductivity. In comparison with conventional aerogel family produced via typical mold casting process (Fig. 3g), the printed RF composite aerogels also show the strong competitiveness. Meanwhile, the printed RF composite aerogels surpass the most 3D printing aerogel insulators so far reported (Fig. 3h). Considering pure silica aerogel is the best reported candidate for thermal insulation, the thermal conductivity value of SiO_2_–RF composite aerogel is only slightly higher than that of silica aerogel, which highlights the major advantages of the superior combination of low thermal insulation and good mechanical properties. Moreover, since the addition of hydrophobic silica aerogel particles, the printed SiO_2_–RF composite aerogels still show hydrophobicity (Fig. S14)**,** the water contact angle is stable over a period of time at 106°, indicating the possible long-term use of the SiO_2_–RF aerogel composite in contact with water or humidity.

### Carbon–SiO_2_ Composite Aerogels and Photothermal Performance

After carbonization and activation at 800 °C, the carbon–SiO_2_ composites change to completely hydrophilic materials (Fig. S14), which is necessary for the following demonstration for solar-driven water evaporation applications. Since the carbon is also activated by CO_2_, we conducted a dynamic vapor sorption (DVS) test to evaluate the water absorption performance. The adsorption capacity curve and kinetic curve of printed aerogels under different relative humidity are shown in Fig. S9. The saturated adsorption capacity of the carbon–SiO_2_ composite is 17.0%, compared with 6.4% for the RF. Additionally, the carbon–SiO_2_ composite exhibits superior adsorption kinetics, reaching adsorption equilibrium faster, demonstrating strong and rapid water absorption performance.

**Fig. 4:**
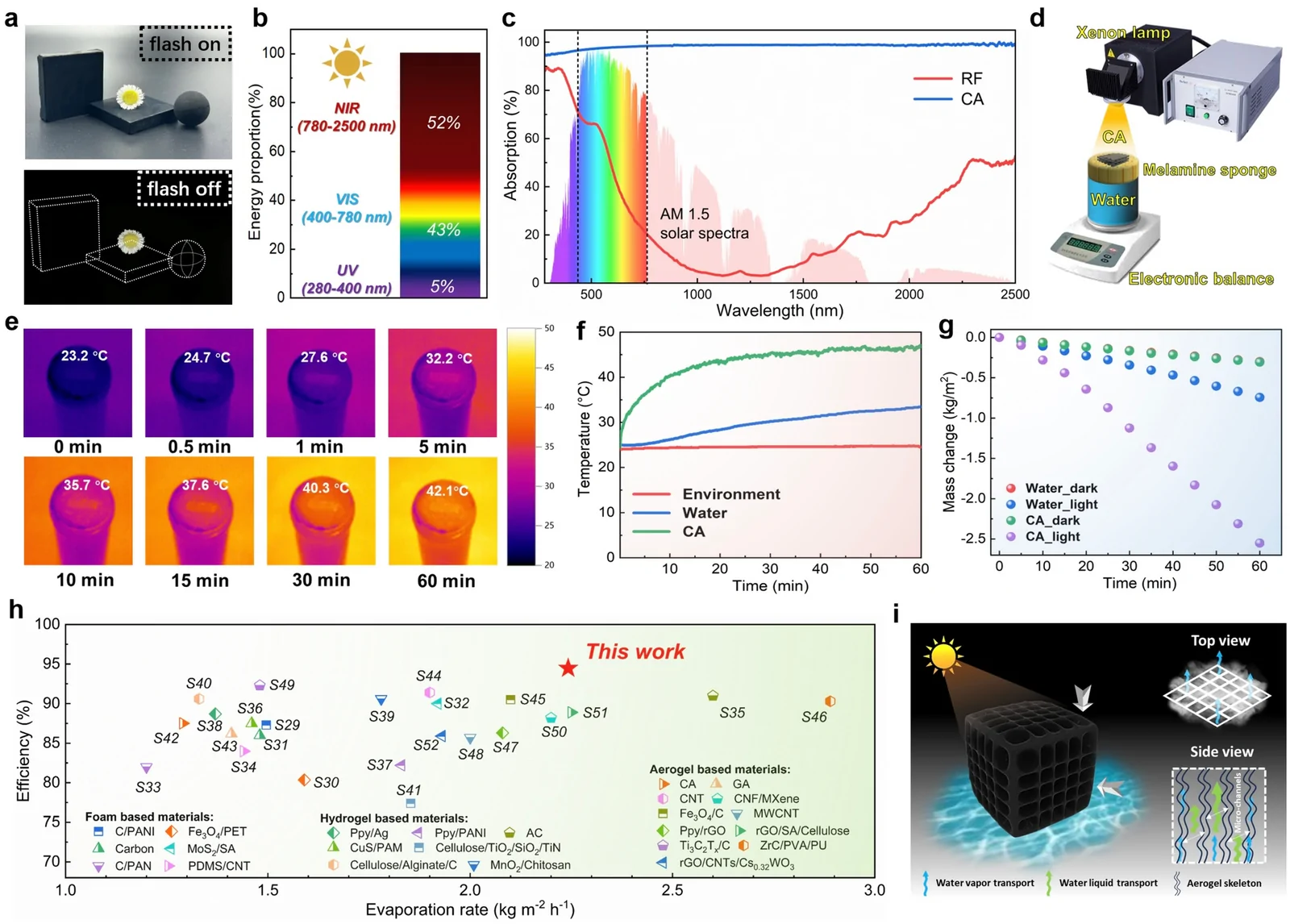
Solar-driven photothermal properties of RF-derived super-black carbon aerogels. **a** Optical photos of super-blacks with and without flash in the daytime, the flower is aim to reflect the position. **b** Specific division of the solar spectrum and their energy proportion. **c** Absorption spectra of SiO_2_–RF and carbon–SiO_2_ composite aerogels across the whole solar spectrum. **d** The schematic diagram of solar-driven evaporation device. **e** IR images of carbon–SiO_2_ under 1.0 sun illumination in humid environment. **f** The carbon–SiO_2_ composite top temperature, water temperature, and ambient temperature changes of evaporators as a function of irradiation time under 1.0 sun illumination. **g** Mass change of water with evaporators as a function of irradiation time under 1.0 sun illumination and dark environment. **h** Comparison of water evaporation rate and photothermal conversion efficiency of carbon–SiO_2_ composite and other state-of-art photothermal conversion materials, including foam-based, hydrogel-based, and aerogel-based materials (Tables S4). **i** Photothermal mechanism based on 3D patterning micro-channels

Figure 4a shows that the shape of the carbon–SiO_2_ composites can be clearly observed under the flash, but without the help of the flash, the materials seem like disappearing, only the flower placed on the super can be recognized. This phenomenon indicates that the material has excellent optical invisibility in the visible light field. As far as the solar spectrum, it has a wide range of wavelengths, including ultraviolet (280–400 nm), visible (400–780 nm), and near-infrared (NIR, 780–2500 nm), with energy shares of 5%, 43%, and 52% [50], respectively (Fig. 4b). Most importantly, as expected, the carbon–SiO_2_ composite aerogel shows an ultra-black feature across the whole solar spectrum. Figure 4c shows the absorption curves of the SiO_2_–RF and the carbon–SiO_2_ across the whole solar spectrum of 280–2500 nm. The diffuse reflectance and transmittance spectra are provided in Figs. S15 and S16. Transmittance was negligible (< 0.1%) across the whole solar band (280–2500 nm), confirming the material’s opacity. Notably, the average absorption of the carbon composite in this range is 99.56%, indicating an ultra-high light absorption performance, demonstrates considerable competitiveness in the previous studies [51, 52]. This ultra-black property is ideal for photothermal conversion due to its ultra-low reflection, ultra-high absorption, and lack of transmission, making it perfect for photothermal applications. As shown in Fig. S17, the carbon–SiO_2_ composite demonstrated a rapid photothermal response, with the top surface temperature rising above 80 °C within 1 min under 1.0 sun illumination, while the RF remained at 40 °C for a longer period. Moreover, the surface temperature quickly dropped to room temperature within a few min after the light source was turned off, indicating a rapid photothermal response of the carbon–SiO_2_ composite. In short, carbon–SiO_2_ with excellent solar absorption capacity and photothermal response shows immense potential in solar evaporation applications.

To evaluate the solar-driven water evaporation performance of carbon–SiO_2_ composite aerogels, we constructed a solar-driven water evaporation device, as shown schematically in Fig. 4d. Melamine foam was used as both a water delivery channel and a thermal insulation layer to prevent heat transfer from the carbon aerogel to the water and to minimize potential heat loss. According to Fig. 4e, the top surface temperature of the carbon–SiO_2_ composite aerogel increased significantly from 23.2 to 27.6 °C within 1 min under 1.0 sun radiation, reaching a maximum temperature of 42.1 °C after 60 min in a humid environment. The changes in the aerogel’s top temperature, water temperature, and ambient temperature as a function of irradiation time under 1.0 sun are recorded in Fig. 4f. The results show that under 1.0 sun illumination, the top temperature of the evaporator reaches an equilibrium of 45 °C within 20 min, demonstrating a temperature difference (ΔT) of 15 °C between the water and 25 °C between the environments. This indicates that the device is an efficient energy conversion evaporator. During water evaporation, the hydrophilic nature of the carbonized carbon–SiO_2_ composite aerogel allows for continuous and rapid water transport from the bottom of the carbon aerogel to the top surface for evaporation. The porous structure of the 3D-printed carbon aerogel provides ample space for water vapor to escape, and its large specific surface area offers numerous sites for water evaporation. The mass changes of pure water and the carbon aerogel membrane under 1.0 sun illumination and in a dark environment over 60 min are shown in Fig. 4g. The evaporation rate of both pure water and the carbon–SiO_2_ composite aerogel in a dark environment were only 0.29 kg m^−2^ h^−1^. However, the real evaporation rate for the printed carbon aerogel reached 2.25 kg m^−2^ h^−1^, with an energy conversion efficiency of 94.2%. Compared to state-of-the-art photothermal conversion materials (Fig. 4h, listed in Table S4), including foam-based, hydrogel-based, and aerogel-based materials, the printed carbon–SiO_2_ composite shows leading performance in terms of evaporation rate and photothermal conversion efficiency. As vividly depicted in Fig. 4i, this impressive result is attributed to the well-controlled 3D structures and micro-channels, whose hierarchical microscale and nanoscale structures allow for rapid directional water transport. The combination of ultra-black wide absorption bands, organized 3D printing micro-channels, and large surface area and pore volume makes the printed carbon–SiO_2_ composite highly competitive for efficient photothermal conversion evaporators.

### Electromagnetic Wave Absorption Performance of the 3D Featured Carbon–SiO_2_ Composite Aerogels

**Fig. 5:**
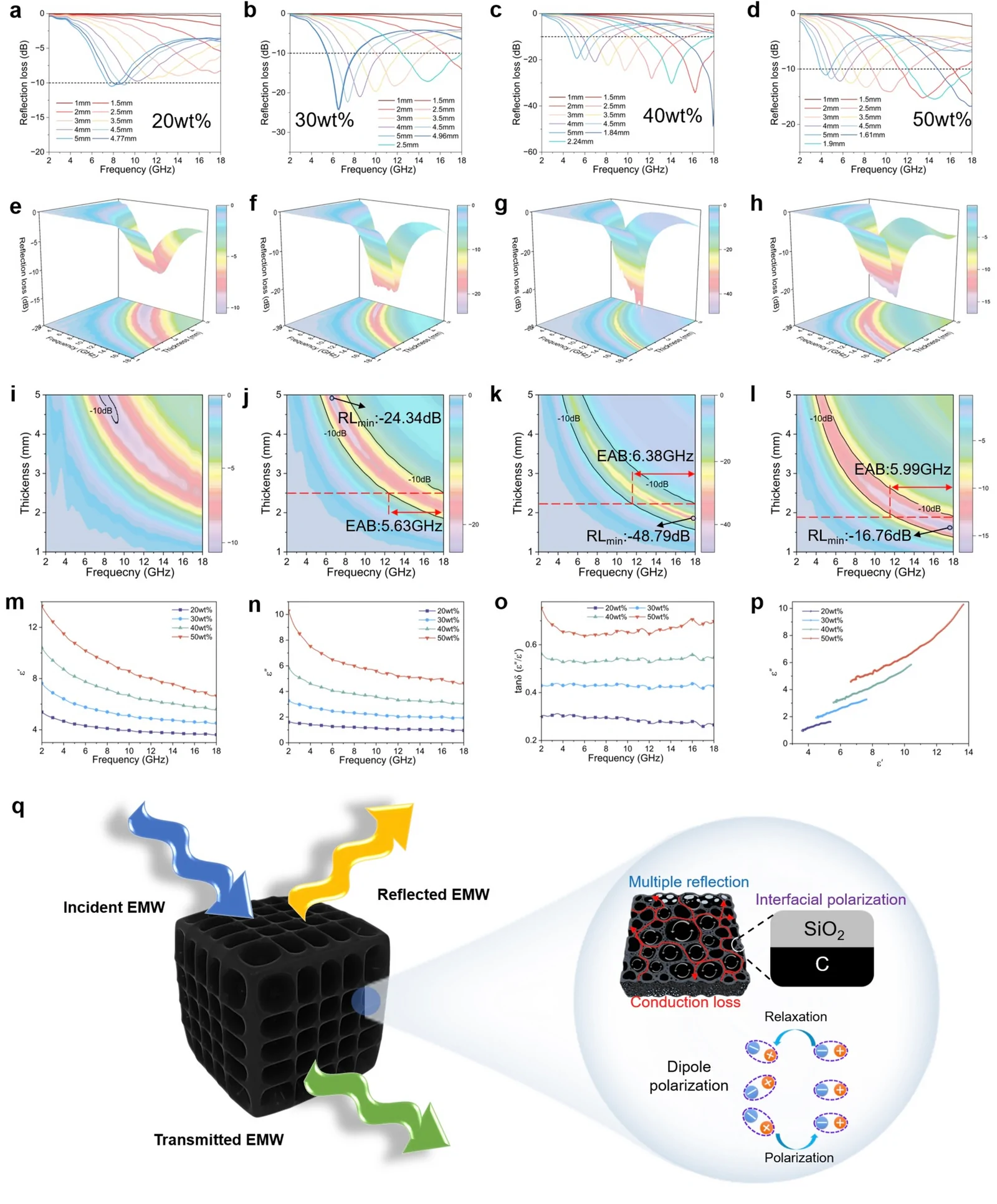
Electromagnetic wave absorption performance of 3D featured carbon–SiO_2_ composite aerogels. **a-d** Two-dimensional plots of RL curves for CAs with different filling contents: **a** 20 wt%, **b** 30 wt%, **c** 40 wt%, and **d** 50 wt%. **e-l** Three-dimensional plots of RL curves for CAs with different filling contents: **e, i** 20 wt%, **f, j** 30 wt%, **g, k** 40 wt%, **h, and l** 50 wt%. **m** Real parts of the complex permittivity for CAs. **n** Imaginary parts of the complex permittivity for CAs. **o** Dielectric loss tangent (tanδ = ε′′/ε′) for CAs. **p** Cole–Cole curves for CAs. **q** Schematic illustration of the EMWA mechanism of CAs

The proper electrical conductivity (Fig. S18) and super-black performance play a crucial role in electromagnetic wave (EMW) energy dissipation. Therefore, we proceed to evaluate its microwave absorption properties. Electromagnetic wave absorption (EMWA) performance is typically considered effective when the reflection loss (RL) falls below -10 dB, corresponding to > 90% attenuation of incident radiation [53]. Figure 5a-l presents 3D surface plots and 2D contour maps of the CAs with varied filler loadings (20 wt%–50 wt%). The 20 wt% composite exhibits limited absorption capability, demonstrating a minimum RL (RL_min_) of -10.51 dB at 7.88 GHz and an effective absorption bandwidth (EAB, RL ≤ -10 dB) of merely 1.0 GHz. Increasing the filler content markedly enhances EMW attenuation: The 30 wt% composite achieves an RL_min_ of -24.34 dB at 6.56 GHz with an EAB of 5.63 GHz at a minimal matching thickness of 2.5 mm. Notably, the 40 wt% composite delivers optimal performance, attaining an exceptional RL_min_ of -48.79 dB at 17.92 GHz and an ultra-wide EAB of 6.38 GHz at a thickness of 2.24 mm. Further increasing the filler to 50 wt%, however, degrades absorption (RL_min_ reaches -16.76 dB at 17.8 GHz; EAB attains 5.99 GHz at 1.9 mm), indicating non-monotonic dependence on filler concentration. Crucially, the RL_min_ peak shifts toward lower frequencies with increasing absorber thickness, consistent with quarter-wavelength destructive interference principles. This frequency-thickness correlation further validates the impedance matching mechanism in these hierarchical aerogels [54]. The EMWA performance is further evaluated with the electromagnetic parameters such as complex permittivity and permeability. The real permittivity (ε′) and permeability (μ′) quantify electric and magnetic energy storage, respectively, while their imaginary components (ε″, μ″) represent energy dissipation [55]. As frequency increases from 2 to 18 GHz (Fig. 5m, n), both ε′ and ε″ of CAs decrease. This frequency-dependent permittivity reduction implies conductive loss as the dominant mechanism. The dielectric loss tangent (tan δ = ε″/ε′), plotted in Fig. 5o, reveals multiple resonance peaks, indicating several polarization relaxations in CAs. Cole–Cole curves based on Debye theory (Fig. 5p) exhibit two distinct regions: multiple semicircles with low ε′ suggest polarization loss, while a linear trend with higher ε′ signifies conduction loss. Mechanistic analysis (Fig. 5q) reveals that heterogeneous interfaces between distinct phases [56] (amorphous carbon and SiO_2_ in the CAs) generate multiple interfacial polarizations, leading to dual-band absorption characteristics. This interfacial engineering synergistically enhances conductive loss, dielectric loss, and polarization relaxation. Consequently, EMW energy dissipation is dominated by the synergistic effects of these multi-scale loss mechanisms.

This unique combination of non-conductive silica particles and conductive carbon matrix results in a material with stable thermal performance, high light absorption, and excellent EMWA ability. In summary, the super-black silica-based nanocomposite aerogels hold great potential for applications in thermal protection, precision optics, and solar energy collection, providing enlightening insights for the multifunctional applications of super-black materials.

## Conclusions

In summary, the incorporation of silica into resorcinol–formaldehyde aerogel significantly enhances their hydrophobicity, thermal insulation, and thermal stability. By adjusting the silica loading, the RF–silica sol can achieve optimal rheology for ink formulation in 3D printing. The printed SiO_2_–RF green bodies, after drying, exhibit excellent mechanical properties and ultra-low superinsulating thermal conductivities (15.8 mW m^–1^ K^–1^). These properties make them attractive as robust thermal insulators. After pyrolysis, the printed SiO_2_–RF composites transform into carbon–silica composite aerogels, showing minimal shrinkage and retaining their 3D-printed features. With a high silica aerogel loading of over 75 wt%, the carbon–SiO_2_ composites exhibit ultra-black properties (> 99.5% absorbance) across a wide frequency range of 280–2500 nm wavelength. These materials demonstrate rapid photothermal response and high solar-driven water evaporation performance with high-efficient energy conversion (evaporation rate 2.25 kg m^−2^ h^−1^ with 94.2% energy efficiency). Besides, the carbonized composite aerogels exhibited an excellent microwave absorption performance of high reflection loss (RL_min_ = – 48.79 dB) and wide bandwidth (EAB_max_ = 6.38 GHz). Benefiting from the 3D patterning technology and the super-black feature, these nanocomposite aerogels open up transformative possibilities for applications in thermal protection, precision optics, solar energy harvesting, and electromagnetic wave absorption.

## Supplementary Information

Below is the link to the electronic supplementary material.

Supplementary file1 (MP4 5038 KB)

Supplementary file2 (MP4 3889 KB)

Supplementary file3 (DOCX 31,636 KB)
