# Supplementary material for: Three-dimensional Patterning Super-Black Silica-Based Nanocomposite Aerogels
Source: Nanomicro Lett. 2025 Aug 20;18:36. doi: 10.1007/s40820-025-01870-6 (PMC12367637; doi:10.1007/s40820-025-01870-6)
Supplement: Supplementary file 3 — Supplementary file3 (DOCX 31,636 KB) [file 40820_2025_1870_MOESM3_ESM.docx]

Supporting Information for

**Three‑dimensional Patterning Super-Black Silica-Based Nanocomposite Aerogels**

Zhiyang Zhao^1, 2^, Romain Civioc^1^, Wei Liu^2^, Peiying Hu^1^, Mengmeng Li^1^, Fuhao Xu^2^, Robin Pauer^3^, Jiabei Luo^4^, Samuel Brunner^1^, Pawel Ziemianski^1^, Ilia Sadykov^1^, Sandra Galmarini^1^, Yong Kong^2^, Xiaodong Shen^2,*^, Wim J. Malfait^1,*^, Shanyu Zhao^1,*^

^1^ Laboratory for Building Energy Materials and Components, Swiss Federal Laboratories for Materials Science and Technology, Empa, Dübendorf 8600, Switzerland

^2^ College of Materials Science and Engineering, Nanjing Tech University, Nanjing 210009, P. R. China

^3^ Electron Microscopy Center, Swiss Federal Laboratories for Materials Science and Technology, Empa, Dübendorf 8600, Switzerland

^4^ Institute of Environmental Engineering, ETH Zurich, Stefano-Franscini-Platz 3, Zürich, 8093 Switzerland

*Corresponding authors. E-mail: [xdshen@njtech.edu.cn](mailto:xdshen@njtech.edu.cn) (Xiaodong Shen); [wim.malfait@empa.ch](mailto:wim.malfait@empa.ch) (Wim J. Malfait); [shanyu.zhao@empa.ch](mailto:shanyu.zhao@empa.ch) (Shanyu Zhao)

**Supplementary Figures and Tables**


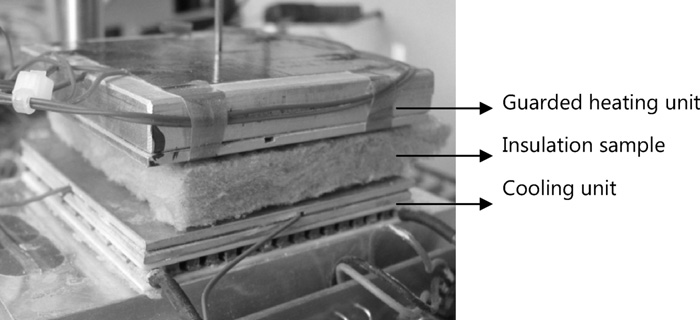


**Fig. S1** A special hot plate device designed for small samples of low thermal conductivity materials is used for thermal conductivity measurements. The sample size used is 50 mm × 50 mm and 10-12 mm in thickness (put in the middle). In order to be consistent with measurements according to the European Standards calibration measurements were carried out using conventional expanded polystyrene samples measured once in the standard test equipment and then cut into smaller pieces to be measured in a second run in the smaller apparatus

**
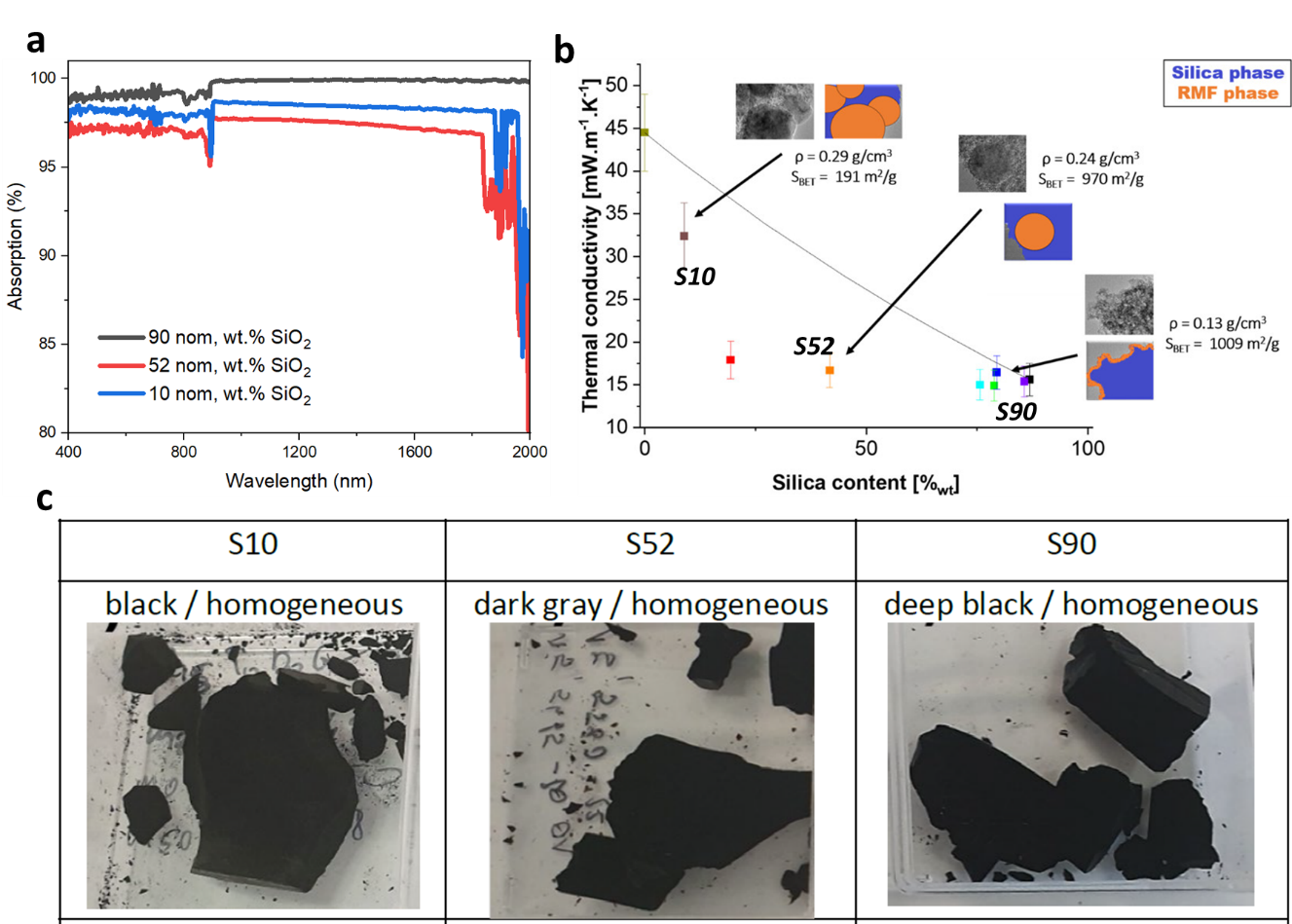
**

**Fig. S2** The relationship between silica content to darkness. **a** Absorption spectra of different silica content carbon-silica composite aerogels. **b** Density and thermal conductivity of different silica content carbon-silica composite aerogels. **c** Appearance of different silica content carbon-silica composite aerogels

**
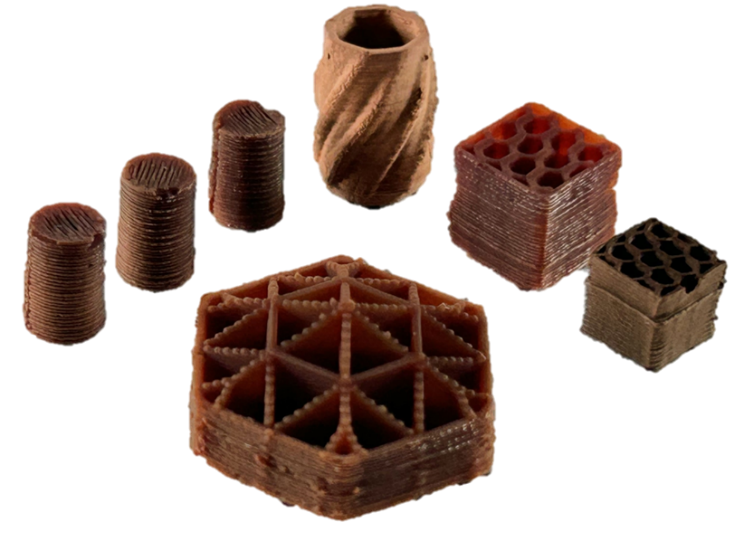
**

**Fig. S3** Optical photos of various 3D patterning SiO_2_-RF composite aerogels printed with different ink recipes listed in Table S1

**
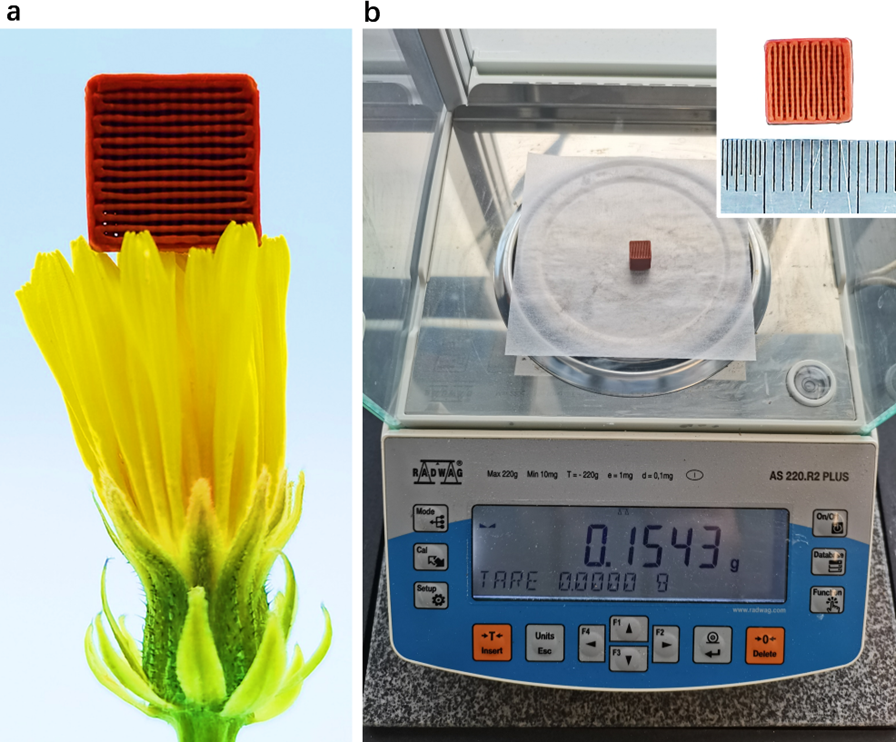
**

**Fig. S4** Lightweight performance of the printed aerogel. **a** Ultralight RF-SiO_2_ aerogel on the flower. **b** Visual weighing test (inset shows the length of the aerogel cube, each small grid represents 0.1 mm)


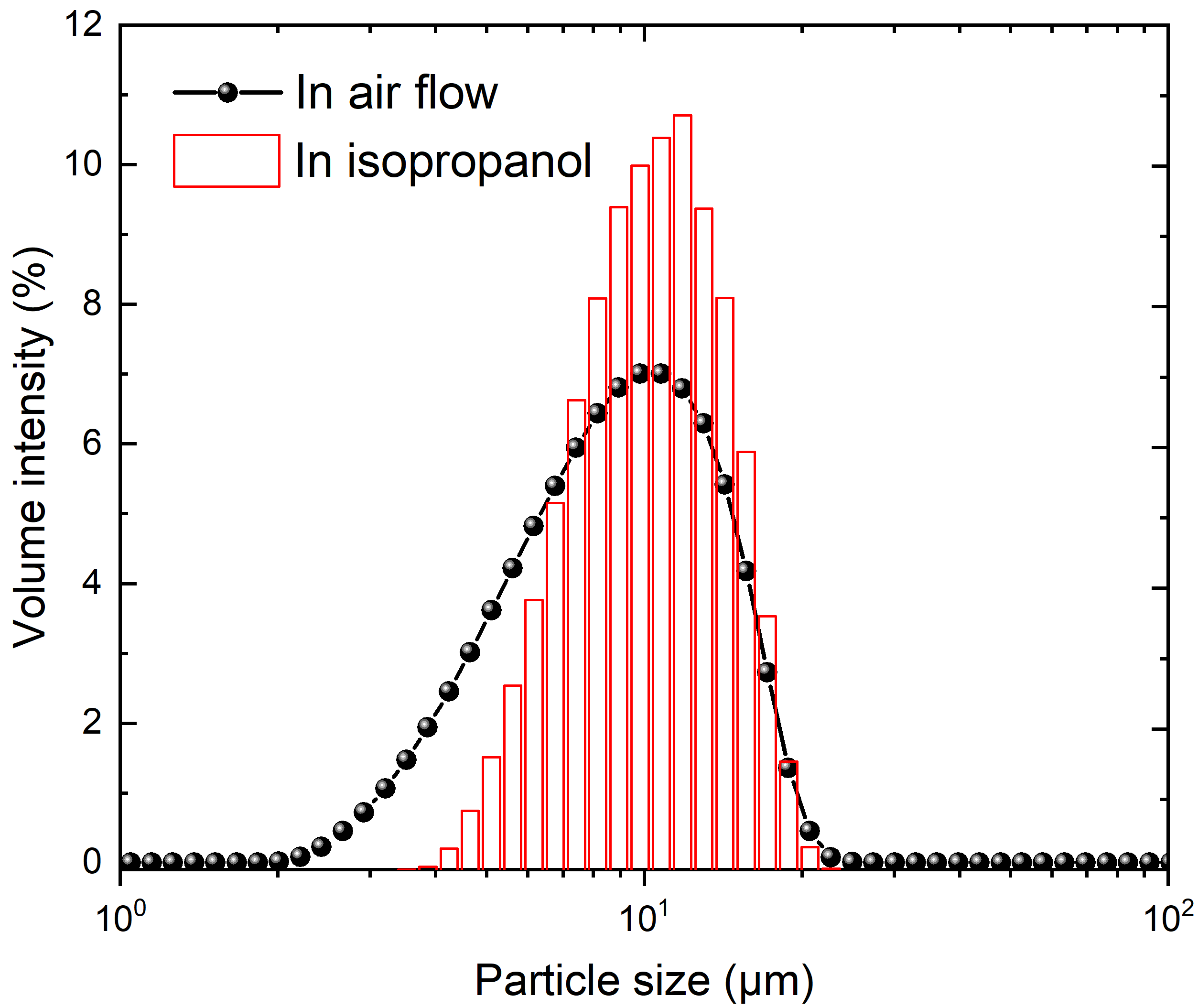


**Fig. S5** Particle size distribution of silica aerogel particles

**
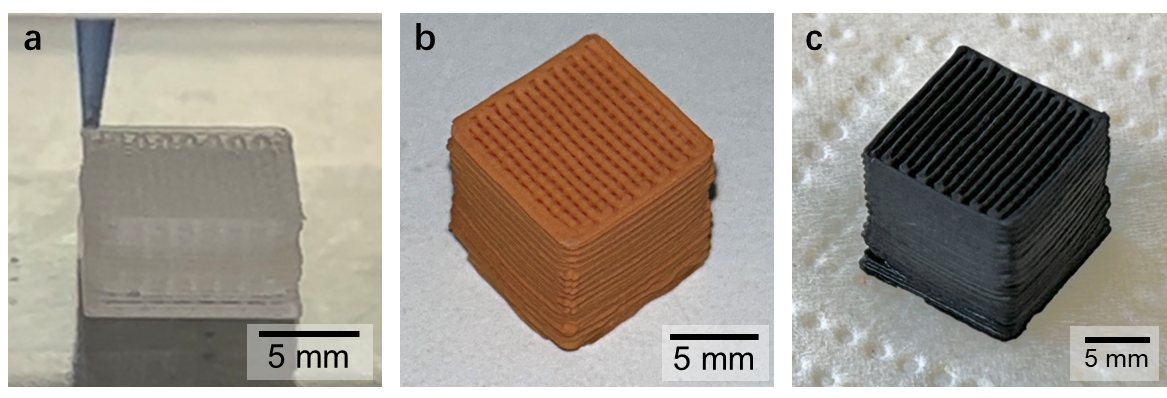
**

**Fig. S6** Optical photos of 3D patterning silica-based nanocomposite aerogels **a** during 3D printing, **b** after ScCO_2_ drying, and **c** after carbonization (printed with SP2.5 ink)


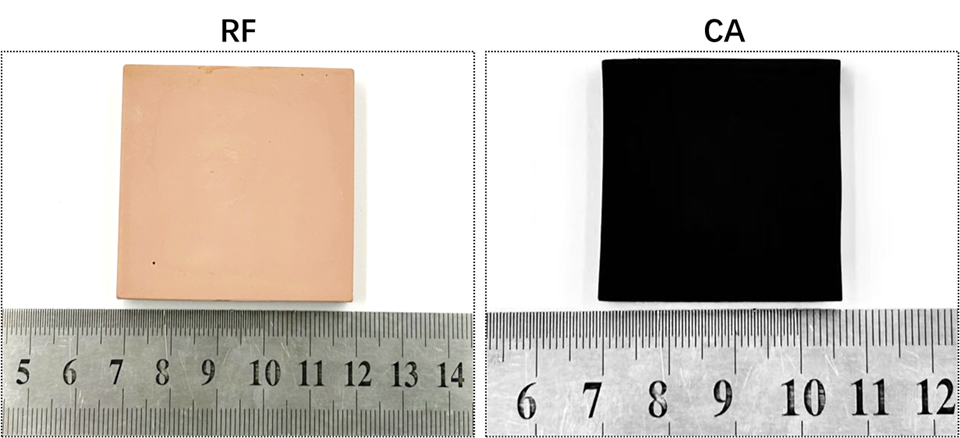


**Fig. S7** The silica-based nanocomposite aerogels with regular dimensions


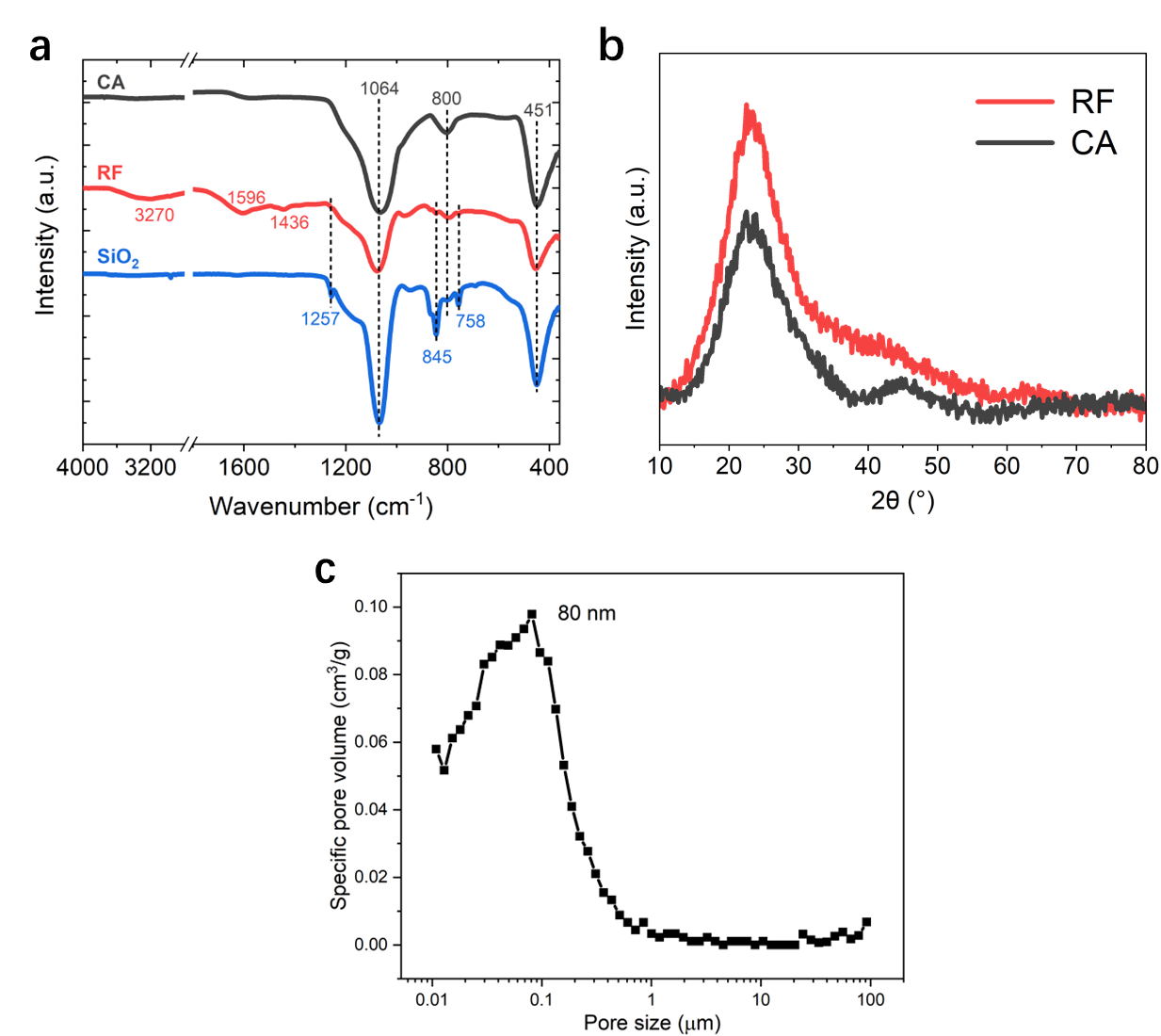


**Fig. S8** Basic characterizations of silica-based nanocomposite aerogels. **a** FT-IR curves. **b** XRD curves. **c** Pore size distribution curve of CA via MIP method


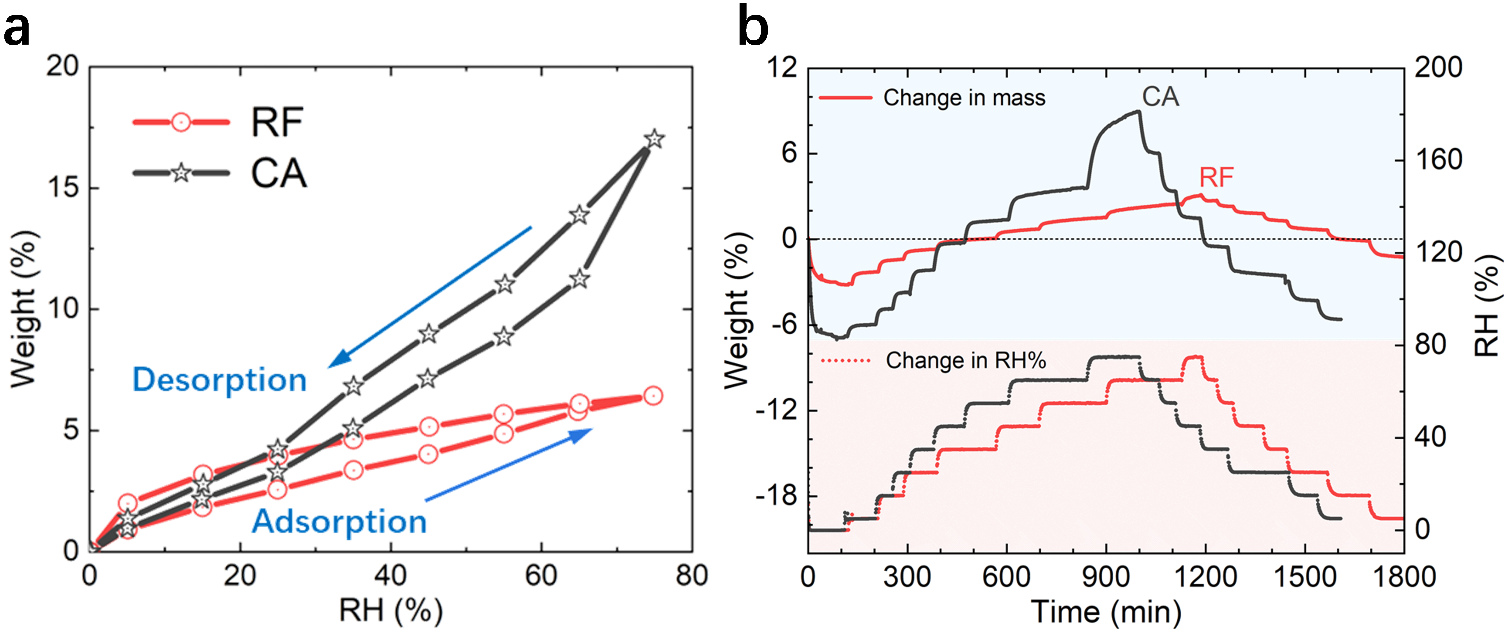


**Fig. S9** Dynamic vapor sorption performance of silica-based nanocomposite aerogels. **a** DVS adsorption and desorption curves, weight of water vapor absorbed in function of relative humidity. **b** Sorption kinetics, the blue region represents change in mass, while the pink represents change in RH%


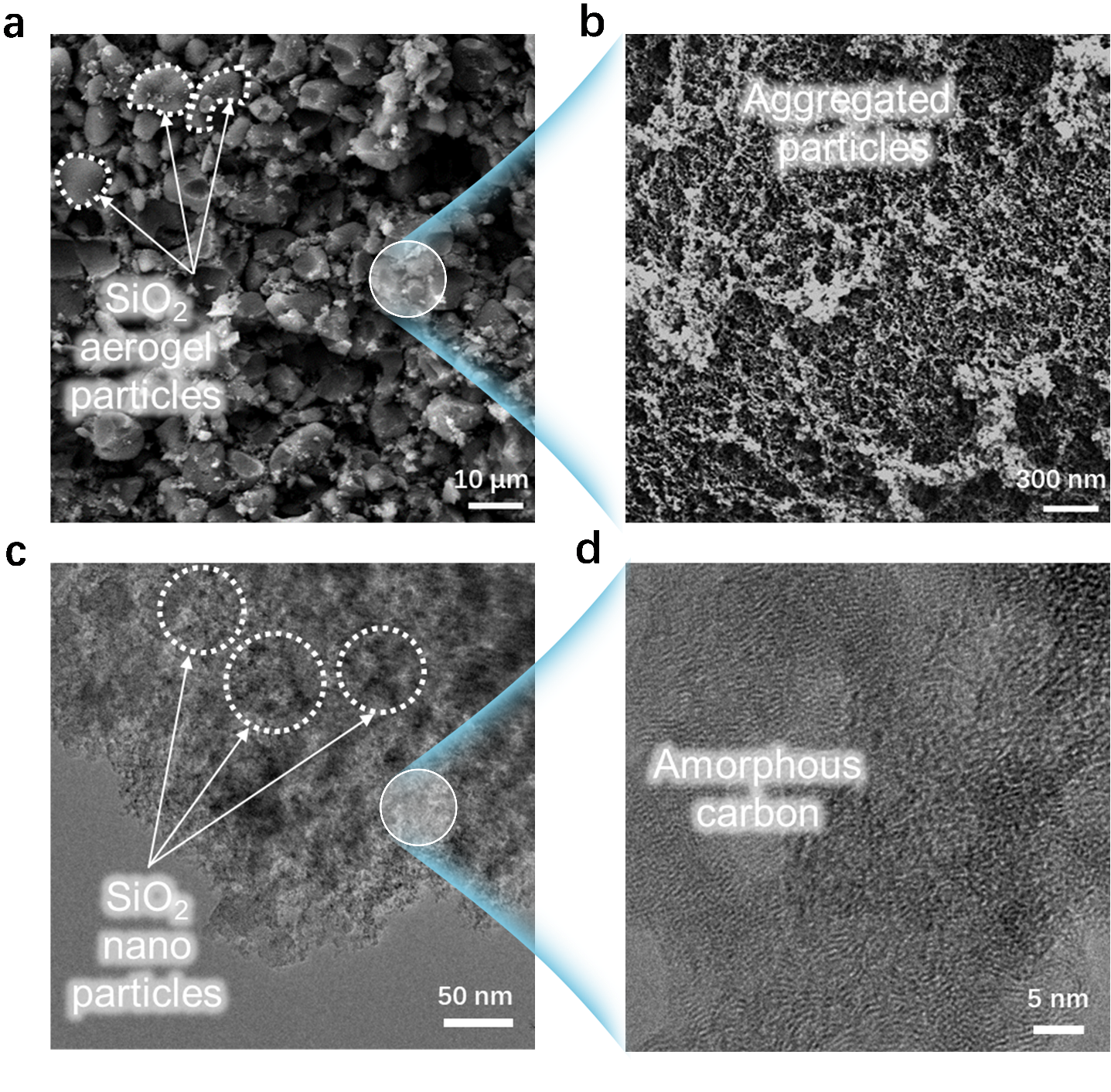


**Fig. S10** Microstructure of silica-based nanocomposite aerogels. **a** Scanning electron microscopy (SEM) images of RF-derived carbon aerogels (from ink SP2.5). **b** Magnification SEM images of RF-derived carbon aerogels (from ink SP2.5). **c** Transmission electron microscopy (TEM) images of SiO_2_-RF derived carbon-SiO_2_ aerogels (from ink SP2.5). **d** Magnification TEM images of RF-derived carbon aerogels (from ink SP2.5)


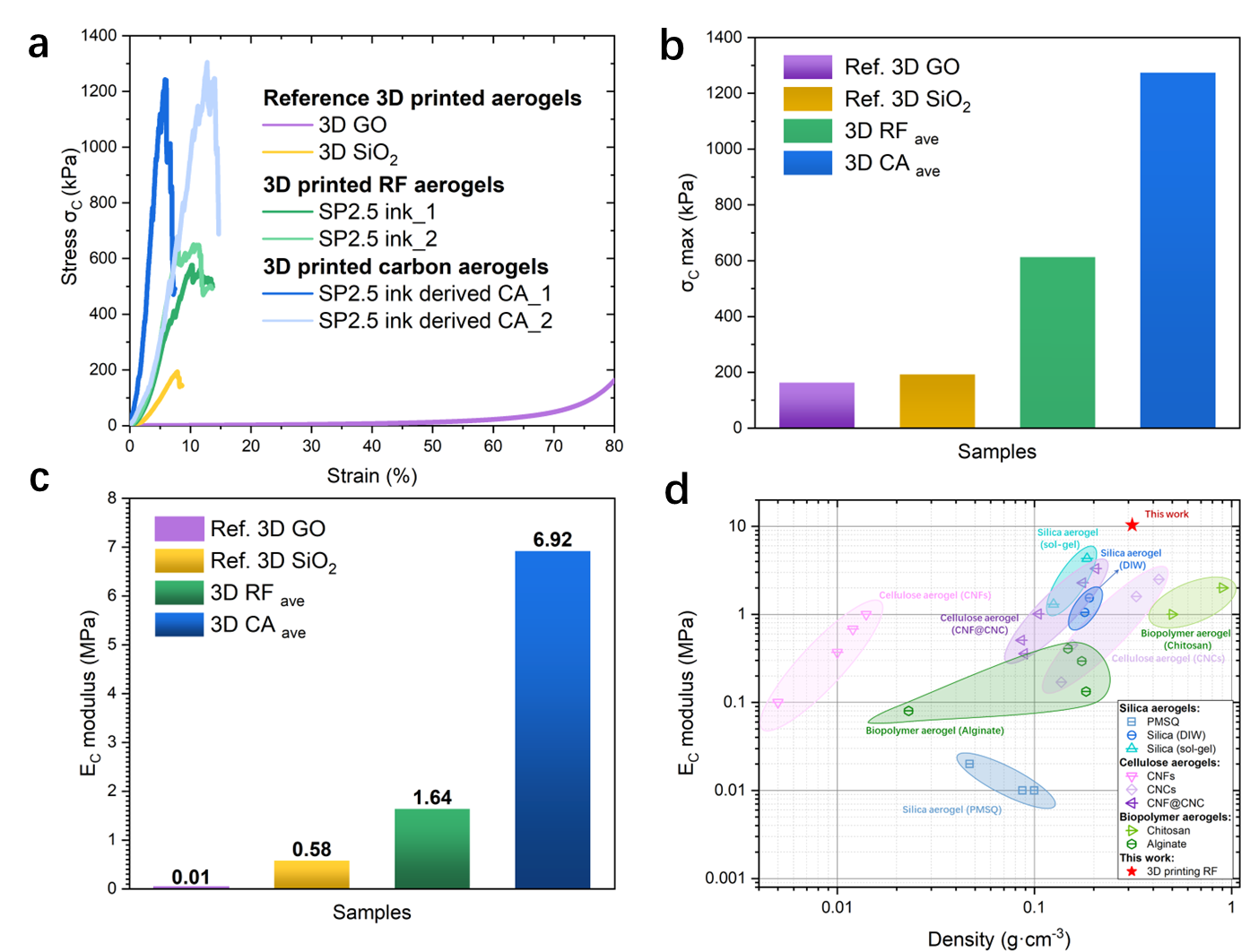


**Fig. S11** Mechanical properties of silica-based nanocomposite aerogels. **a** Stress-strain curves during uniaxial compression of reference 3D patterning aerogels and silica-based aerogels. **b** Maximum stress of reference 3D printed aerogels and silica-based aerogels during uniaxial compression. **c** Young’s modulus of reference 3D printed aerogels and silica-based aerogels during uniaxial compression. **d** Young's modulus values of selected aerogels with low densities


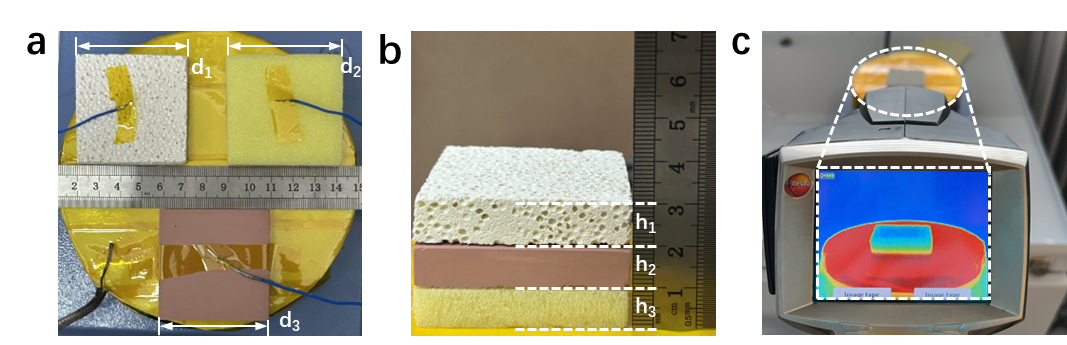


**Fig. S12** Extended data for thermal management test. **a** Physical diagram of the hot stage test of three kinds of insulation materials (SiO_2_-RF composite aerogel, PU foam, and cement brick materials) of the same size (5.0 cm×5.0 cm) and thickness (1.0 cm) from the top view, the thermocouples were tightly attached to the upper surface of the samples, d_1_=d_2_=d_3_=5.0 cm. **b** Side view of the test samples, h_1_=h_2_=h_3_=1.0 cm. **c** Use tape to reduce the reflectivity of the metal hot stage


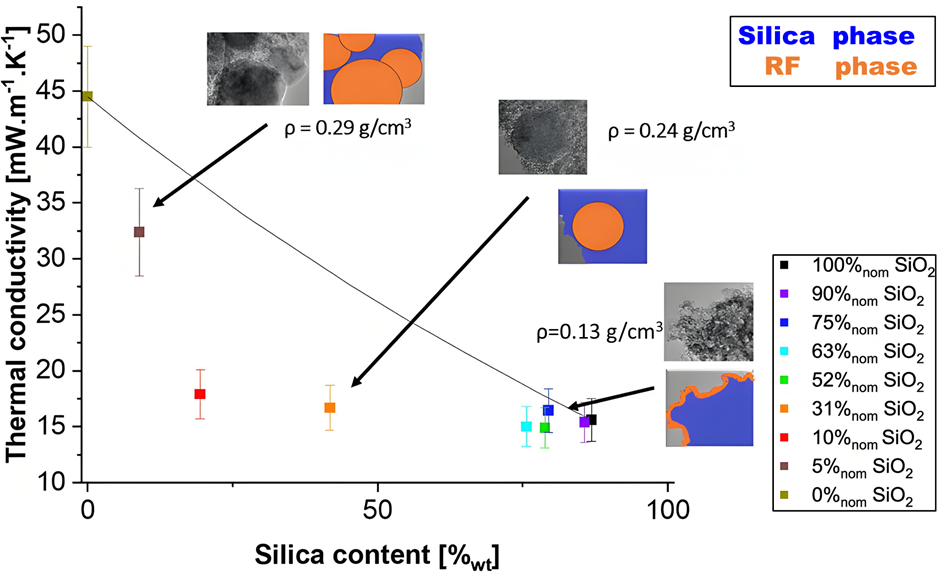


**Fig. S13** Thermal conductivity of composite aerogels with different SiO_2_ content (from 0 to 100%)


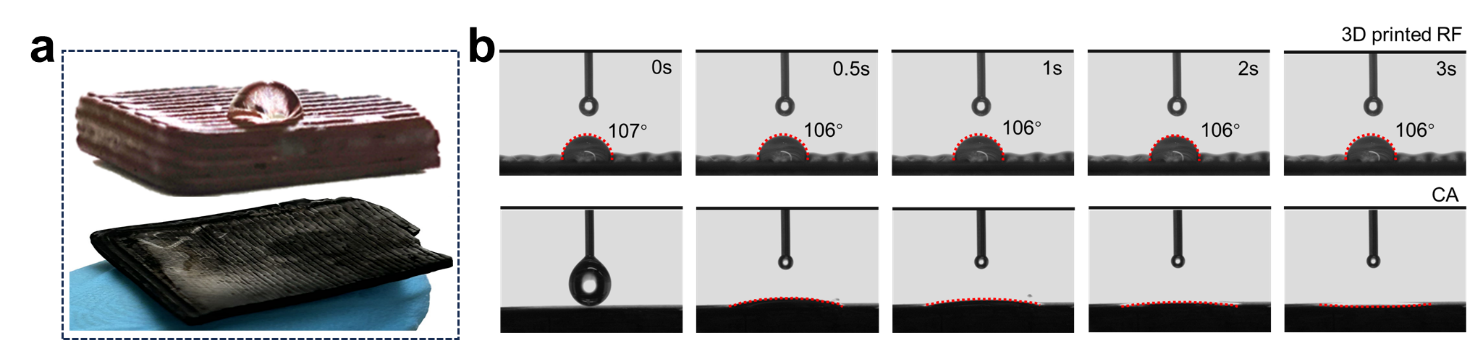


**Fig. S14** Hydrophobic and hydrophilic performance of silica-based nanocomposite aerogels. **a** Optical photos of water droplets on SiO_2_-RF and carbon-SiO_2_ composite surfaces, the water droplets cannot maintain on the carbon-SiO_2_ composite surface due to the hydrophilicity. **b** Water contact-angle measurements of SiO_2_-RF and carbon-SiO_2_ composite aerogels


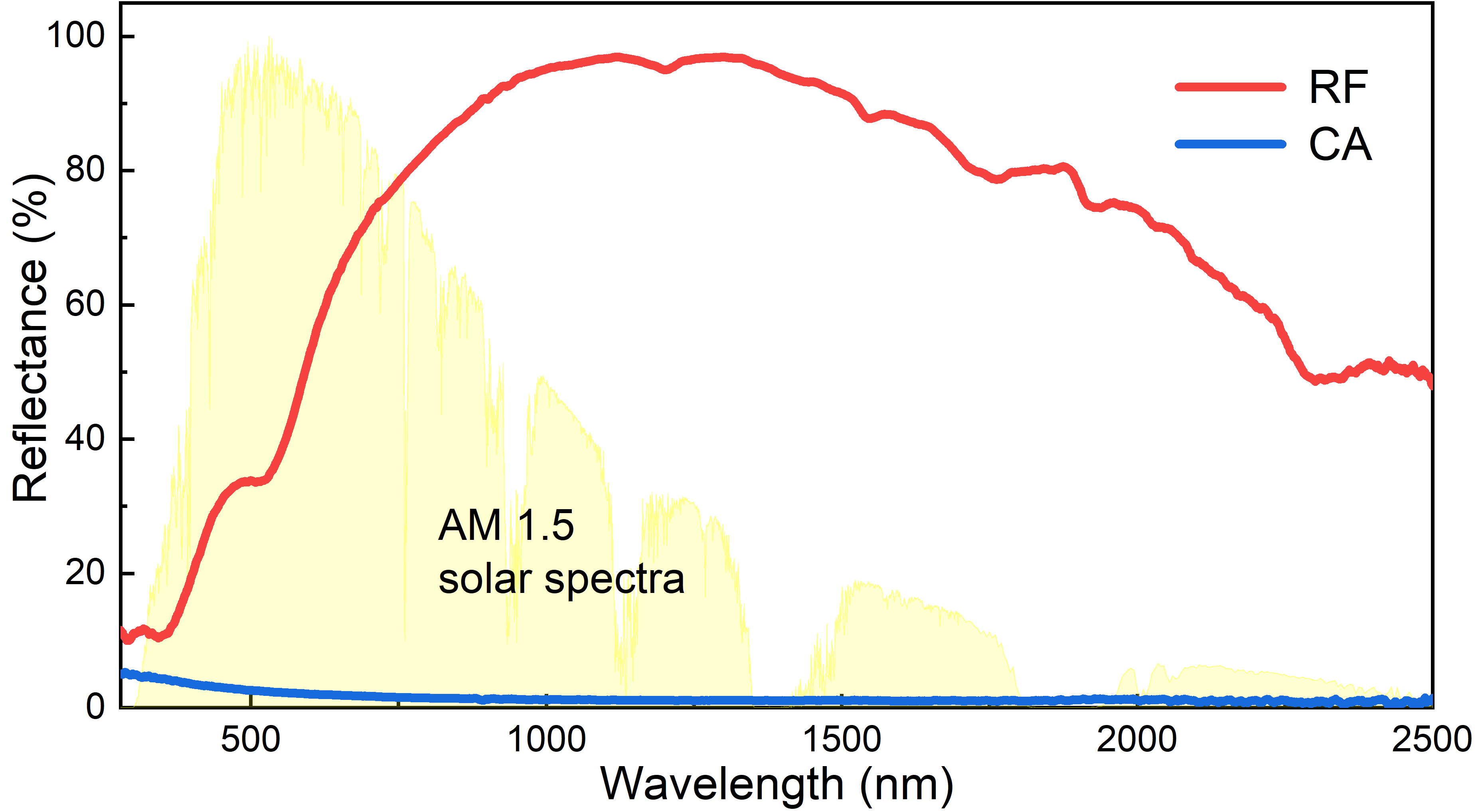


**Fig. S15** Diffuse reflectance spectra of silica-based nanocomposite aerogels in the solar spectral range (280-2500 nm)


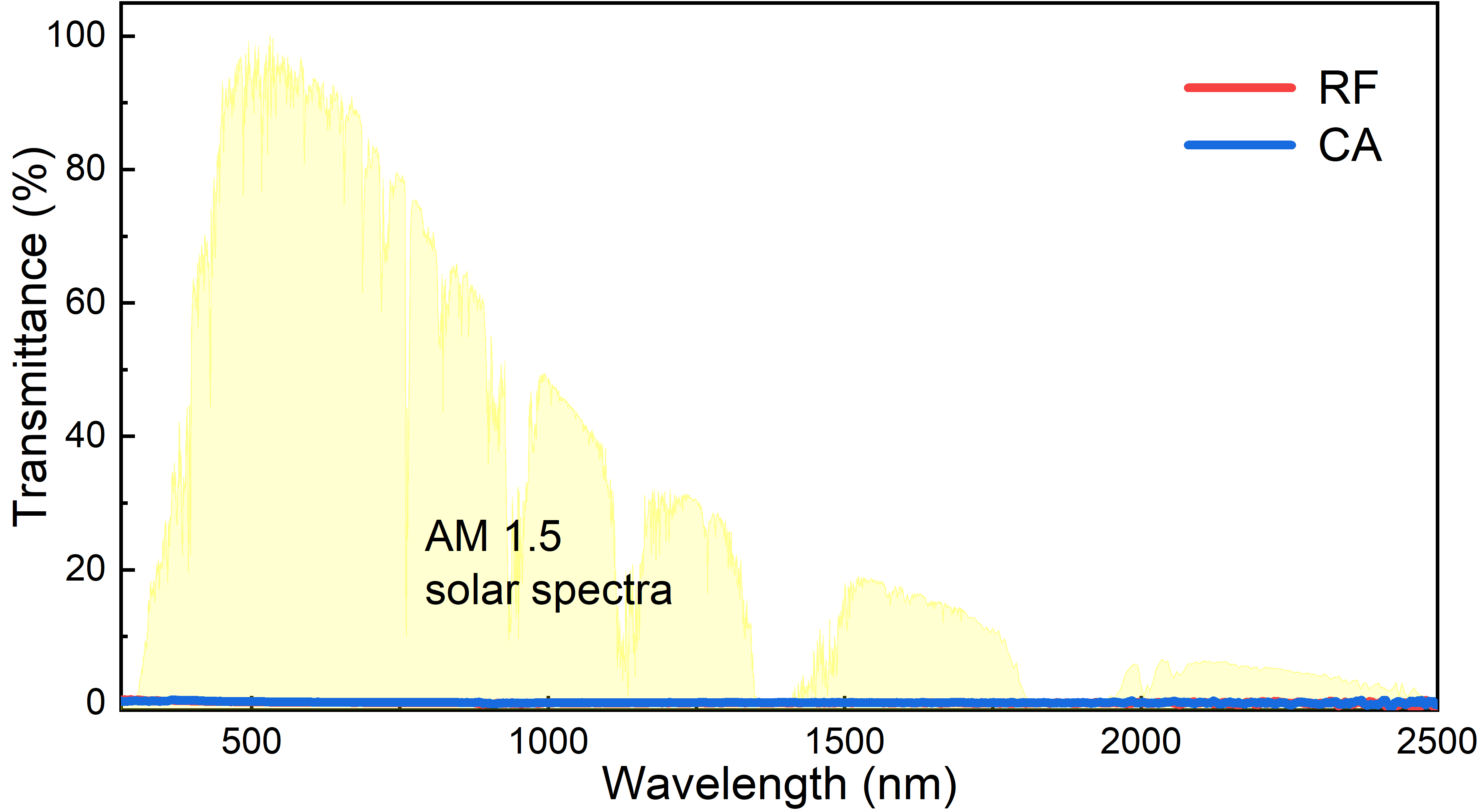


**Fig. S16** Transmittance spectra of silica-based nanocomposite aerogels in the solar spectral range (280-2500 nm)


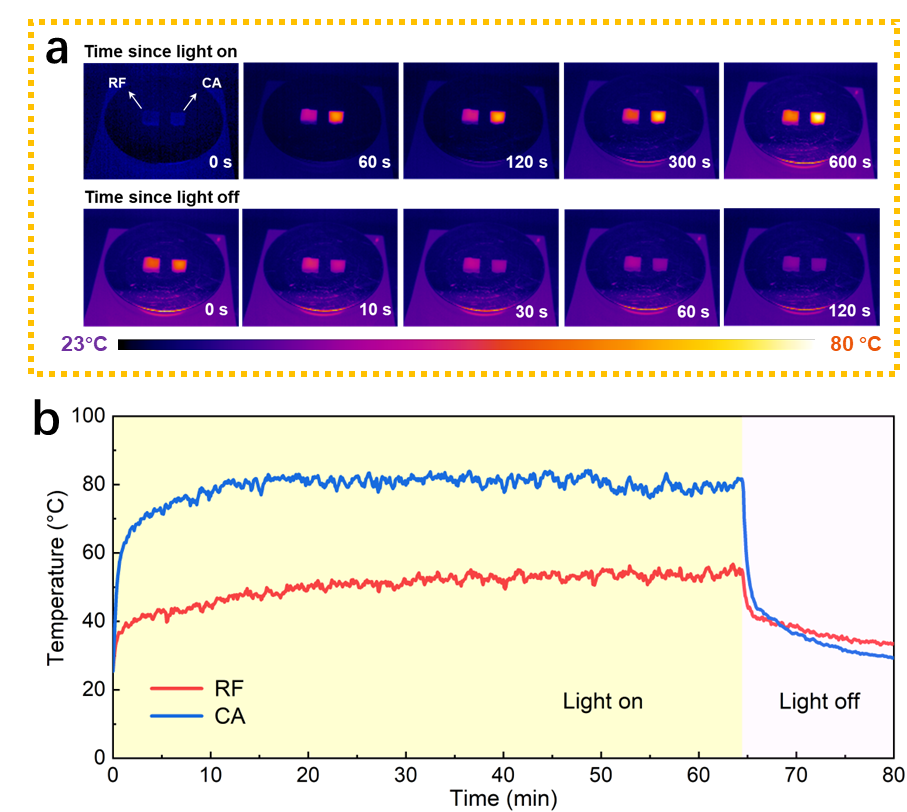


**Fig. S17** Photothermal response performance under dry environment. (a) IR images of 3D patterning SiO_2_-RF aerogel and carbon-SiO_2_ aerogel under 1.0 sun illumination in the dry environment. (b) Temperature response curves of silica-based nanocomposite aerogels

**Electrical Performance**


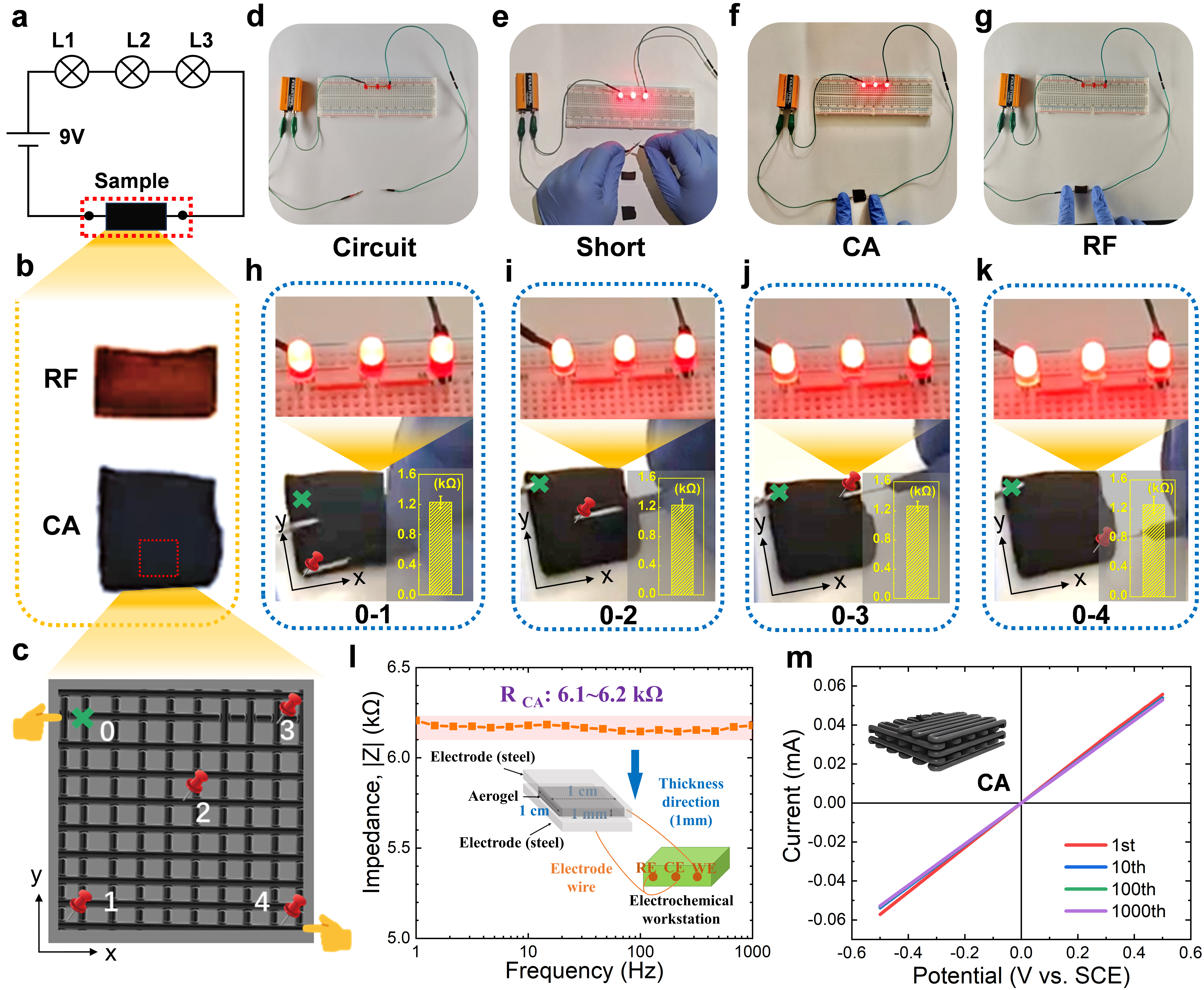


**Fig. S18** Electrical performance of 3D featured carbon-SiO_2_ composite aerogel. **a** Physical diagram of a designed circuit. **b** Optical photos of different printed aerogel samples. **c** Magnification of the red-boxed region in **b**, showing the simplified route model on 3D printed carbon-SiO_2_ composite aerogel surface, one wire is always fixed at 0, and the other wire is connected from point 1 to 4. **d** Physical diagram of a circuit. **e** Short connect circuit diagram. **f** A circuit constructed with carbon-SiO_2_ composite aerogel. **g** A circuit constructed with SiO_2_-RF composite aerogel. **h-k** Conductive stability via different paths from **h** 0-1, **i** 0-2, **j** 0-3, and **k** 0-4, insert is the column of corresponding resistance detected by the ohmmeter, showing the path-independent resistance performance. **l** Electrical impedance of Bode plot in the thickness direction of carbon-SiO_2_ composite aerogel (inset: testing method). **m** Cyclic voltammograms of carbon-SiO_2_ composite aerogel at a scan rate of 20 mV s^-1^ in a dry two-electrode system

Apart from their intricate spatial structures, the introduction of non-conductive phases during printing process might have dominant influence on conductivity performance. To better understand the conductive path of the carbonized composites and their 3D structured features, we developed a circuit to test the obtained carbon-SiO_2_ composite (Fig. S18a). Conductive clips were attached to the sample (Fig. S18b) and connected to LED bulbs. The circuit detection shows that the carbon composite is conductive, whereas the RF composite is completely non-conductive (Figs. S18 d-g). 3D printing can create a conductive pattern that facilitates mass transfer by reducing electron diffusion resistance. To better illustrate this, a simplified 2D plane coordinate system (*x, y*) of the 3D printed aerogel is shown in Fig. S18c. The brightness of the LED bulbs visually displays the resistance changes of carbon-SiO_2_ under different specific paths from 0-1, 0-2, 0-3, and 0-4, corresponding to the lattice path in Fig. S18c. We also tested the resistance values under different specific paths (Figs. S18h-k and Movie S2) using the probe contact method via a multimeter. The corresponding resistance values are all 1.2 ± 0.1 kΩ, indicating the path-independent resistance performance. For electrochemical performance, the Bode impedance (Z) in Fig. S18l is expressed as electrical conduction in the thickness (T) direction of a rectangular body with cross-section dimensions (length L, width W) and thickness T. The impedance of the carbon-SiO_2_ composite membrane with 1mm thickness shows a stability of 6.1-6.2 kΩ at a wide frequency range of 1 to 1000 Hz. The carbon-SiO_2_ composite does not show any supercapacitor potential, as displayed in the cyclic voltammetry curves (Fig. S18m), due to the addition of non-conductive silica. Yet, it exhibits stable voltammograms cycle performance, which means this material is stable under electrical condition, and can be used for any case that need some conductivity but not change with external conditions.


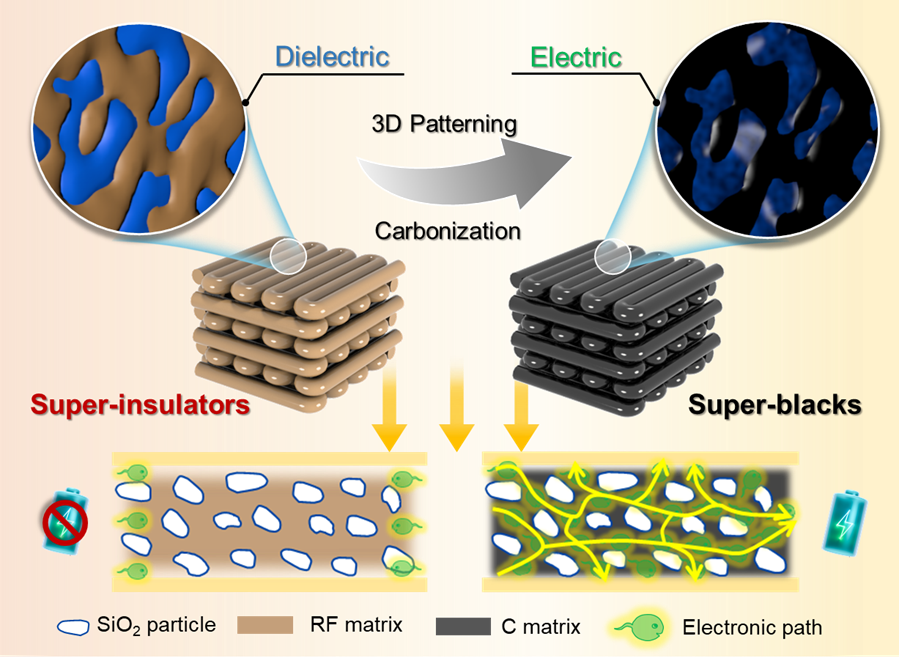


**Fig. S19** Conductive mechanism of super-black silica-based nanocomposite aerogels

For the super-insulation material, both the RF matrix and SiO_2_ particles are non-conductive. In consequence, electrons cannot be transported, result in no conductivity path inside. As for the super-black material, it is composed of conductive carbon matrix and non-conductivity silica. As vividly shown in Fig. S19, despite the transportation of electrons might encounter difficulties when faced with non-conductivity SiO_2_ particles. They can still flow smoothly through the abundant conductive carbon layer and thus form various conductive paths. The introduction of the non-conductive phase, combined with the uniformity of 3D printing, enables the integration of non-conductive microphases and conductive pathways.

**Table S1** SiO_2_-RF composite aerogel ink compositions and properties

| Inks | R | F | 1-Pentanol | P750P20 (PP) | SP ^a^ | PPGNH | ρ |
| --- | --- | --- | --- | --- | --- | --- | --- |
|  | g | ml | | | g | | g cm^-3^ |
| SiO_2_ ink | - | - | 26 | 5 | 5.0 | 4 | 0.22±0.02 |
| SP5.0 ink | 0.7 | 7.5 | 26 | 5 | 5.0 | - | 0.28±0.03 |
| SP2.5 ink | 0.7 | 1.5 | 26 | 2.5 | 2.5 | - | 0.16±0.02 |
| SP2.5PP0 ink | 0.7 | 1.5 | 26 | 0 | 2.5 | - | 0.12±0.05 |

^a.^ SP, refers to SiO_2_ aerogel particle purchased from Cabot Corporation.

**Table S2** Synthesis conditions used for the preparation of the different silica-RMF alcogels, prior to gelation. The second line represents the molar percentage of silica species over silica plus RMF species, while the third line represents the same ratio in weight percentage

| Name | S100 | S90 | S75 | S63 | S52 | S31 | S10 | S05 | RMF ^a^ |
| --- | --- | --- | --- | --- | --- | --- | --- | --- | --- |
| ^b^ SiO_2_ %_nom_ | 100 | 90.0 | 74.8 | 63.3 | 52.1 | 30.9 | 10.0 | 5.0 | 0 |
| ^c^ SiO_2_ %_nom,wtc_ | 100 | 90.4 | 75.6 | 64.3 | 53.2 | 31.9 | 9.6 | 4.8 | 0 |
| P750 Sol (mL) | 40.0 | 39.4 | 38.2 | 37.0 | 35.4 | 25.2 | 17.7 | 8.0 | 0 |
| RMF Sol (mL) | 0 | 0.6 | 1.8 | 3.0 | 4.6 | 9.6 | 22.3 | 32.0 | 40.0 |

^a.^ RMF, Resorcinol–Melamine–Formaldehyde

^b.^ The nominal silica content (SiO_2_ %_nom_) can be calculated using the following equations:

${\%}_{nom}({SiO}_{2})=\frac{M({SiO}_{2})}{M\left( {SiO}_{2} \right)+M\left( R \right)+M\left( M \right)+M(F)}$ (S1)

^c.^ The nominal silica weight content (SiO_2_ %_nom, wt_) can be calculated using the following equations:

${\%}_{nom,wt}({SiO}_{2})=\frac{wt({SiO}_{2})}{wt\left( {SiO}_{2} \right)+wt\left( R \right)+wt\left( M \right)+wt(F)}$ (S2)

**Table S3** Thermal conductivity comparison with different state-of-art materials including commercial materials, conventional and 3D printing aerogels

| Materials | Density  (g cm^-3^) | Thermal conductivity  (mW m^-1^ K^-1^) | Refs. | Group |
| --- | --- | --- | --- | --- |
| PU | 0.025 | 30 | [S1] | Commercial |
|  | 0.050 | 20 |  |  |
| EPS | 0.030 | 33 | [S2] |  |
|  | 0.055 | 38 |  |  |
| Mineral wool | 0.06 | 56 | [S3] |  |
|  | 0.08 | 40 |  |  |
|  | 0.10 | 46 |  |  |
|  | 0.12 | 58 |  |  |
|  | 0.20 | 64 |  |  |
| Glass fiber | 0.17 | 44 | [S4] |  |
|  | 0.20 | 50 |  |  |
| PI foam | 0.03 | 35 | [S5] |  |
|  | 0.06 | 60 |  |  |
| Foamed ceramic | 0.30 | 57.9 | [S6] |  |
|  | 0.42 | 82.6 |  |  |
| Foamed cement | 0.40 | 80 | [S7] |  |
|  | 0.57 | 200 |  |  |
| Silica aerogel | 0.184 | 17.8 | [S8] | Conventional |
|  | 0.218 | 19.3 |  |  |
|  | 0.261 | 23.3 |  |  |
| PMSQ aerogel | 0.125 | 41 | [S9] |  |
|  | 0.075 | 38 |  |  |
|  | 0.105 | 36 |  |  |
|  | 0.141 | 44 |  |  |
| RF aerogel | 0.060 | 20.2 | [S10] |  |
|  | 0.074 | 19.6 |  |  |
|  | 0.082 | 16 |  |  |
|  | 0.102 | 16.7 |  |  |
| PI aerogel | 0.114 | 30.9 | [S11] |  |
|  | 0.141 | 31 |  |  |
| PI aerogel | 0.137 | 33.4 | [S12] |  |
|  | 0.124 | 33.6 |  |  |
|  | 0.246 | 66.8 |  |  |
|  | 0.300 | 45.3 |  |  |
| PUA aerogel | 0.354 | 33.5 | [S13] |  |
|  | 0.344 | 41.3 |  |  |
|  | 0.414 | 34.3 |  |  |
| BN aerogel | 0.032 | 36 | [S14] |  |
|  | 0.093 | 32 |  |  |
|  | 0.096 | 31 |  |  |
|  | 0.097 | 31 |  |  |
|  | 0.029 | 25 |  |  |
| SiC aerogel | 0.305 | 34 | [S15] |  |
|  | 0.378 | 49 |  |  |
|  | 0.446 | 58 |  |  |
| Si_3_N_4_ aerogel | 0.192 | 60.7 | [S16] |  |
|  | 0.164 | 49.1 |  |  |
|  | 0.164 | 45.9 |  |  |
| GO aerogel | 0.005 | 21 | [S17] |  |
|  | 0.019 | 28 |  |  |
| Alginate aerogel | 0.015 | 31.5 | [S18] |  |
| Alginate aerogel | 0.036 | 33.2 | [S19] |  |
|  | 0.052 | 37 |  |  |
| Cellulose aerogel | 0.147 | 36.7 | [S20] |  |
|  | 0.199 | 43.2 |  |  |
|  | 0.235 | 50.7 |  |  |
| Cellulose aerogel | 0.024 | 24 | [S21] |  |
|  | 0.033 | 27 |  |  |
|  | 0.023 | 18 |  |  |
| Chitosan aerogel | 0.094 | 24.1 | [S22] |  |
|  | 0.121 | 25.7 |  |  |
|  | 0.144 | 27.9 |  |  |
|  | 0.167 | 30.9 |  |  |
|  | 0.225 | 36.3 |  |  |
| 3D SiO_2_ | 0.17 | 17.4 | [S23] | 3D printing |
|  | 0.18 | 15.9 |  |  |
|  | 0.2 | 17.2 |  |  |
|  | 0.18 | 15.5 |  |  |
|  | 0.22 | 18 |  |  |
| 3D PI/SiO_2_ | 0.135 | 20.3 | [S24] |  |
| 3D Cellulose | 0.0558 | 24.4 | [S25] |  |
|  | 0.065 | 26.5 |  |  |
|  | 0.068 | 26.5 |  |  |
|  | 0.072 | 27.7 |  |  |
|  | 0.09 | 31.2 |  |  |
|  | 0.085 | 33.5 |  |  |
|  | 0.094 | 31.9 |  |  |
|  | 0.089 | 36.7 |  |  |
|  | 0.105 | 45 |  |  |
|  | 0.109 | 48 |  |  |
|  | 0.115 | 42.5 |  |  |
|  | 0.132 | 55.2 |  |  |
|  | 0.152 | 42.5 |  |  |
|  | 0.166 | 50 |  |  |
| 3D SF/SiO_2_ | 0.13 | 33 | [S26] |  |
|  | 0.17 | 39 |  |  |
| 3D Ceramic | 0.157 | 26 | [S27] |  |
| 3D Ceramic | 0.31 | 35 | [S28] |  |
|  | 0.25 | 34.4 |  |  |
|  | 0.21 | 36.2 |  |  |
|  | 0.36 | 35.2 |  |  |
|  | 0.32 | 33.5 |  |  |
|  | 0.3 | 32.6 |  |  |
|  | 0.36 | 45.8 |  |  |
|  | 0.33 | 43.3 |  |  |
|  | 0.3 | 44.6 |  |  |
| 3D patterning aerogel | 0.12 | 15.8 | This work |  |
|  | 0.23 | 25.1 |  |  |

**Table S4** Photothermal conversion efficiency comparison with different state-of-art materials including foam, hydrogel and aerogel materials

| Materials | Evaporation rate | Photothermal efficiency | Ref. |
| --- | --- | --- | --- |
|  | (kg m^-2^ h^-1^) | (%) |  |
| C/PANI | 1.497 | 87.3 | [S29] |
| Fe_3_O_4_/PET | 1.59 | 80.35 | [S30] |
| Carbon | 1.48 | 86 | [S31] |
| MoS_2_/SA | 1.92 | 90 | [S32] |
| C/PAN | 1.2 | 82 | [S33] |
| PDMS/CNT | 1.44 | 84 | [S34] |
| Activated carbon | 2.6 | 91 | [S35] |
| CuS/PAM | 1.46 | 87.5 | [S36] |
| Ppy/PANI | 1.83 | 82.2 | [S37] |
| Ppy/Ag | 1.37 | 88.7 | [S38] |
| MnO_2_/Chitosan | 1.78 | 90.6 | [S39] |
| Cellulose/Alginate/C | 1.33 | 90.6 | [S40] |
| Cellulose/TiO_2_/SiO_2_/TiN | 1.853 | 77.39 | [S41] |
| CA | 1.29 | 87.51 | [S42] |
| GA | 1.41 | 86.2 | [S43] |
| CNT | 1.9 | 91.4 | [S44] |
| Fe_3_O_4_/C | 2.1 | 90.5 | [S45] |
| ZrC/PVA/PU | 2.89 | 90.3 | [S46] |
| Ppy/rGO | 2.08 | 86.3 | [S47] |
| MWCNT | 2.0 | 85.7 | [S48] |
| Ti_3_C_2_T_x_/C | 1.48 | 92.3 | [S49] |
| CNF/MXene | 2.2 | 88.2 | [S50] |
| rGO/SA/Cellulose | 2.25 | 88.9 | [S51] |
| rGO/CNTs/Cs_0.32_WO_3_ | 1.93 | 85.9 | [S52] |
| 3D patterning aerogel | 2.25 | 94.2 | This work |

**Supplementary Notes**

**Note S1** **Calculation of photothermal conversion efficiency**

The evaporation rate ($\nu$) was calculated by following formula [S53]:

$$\nu=dm/St$$

where *m* represented the water mass change during the evaporation, *S* represented the projected area of the carbon aerogel, and *t* represented the evaporation time, respectively.

The energy efficiency (*η*) for solar-vapor conversion could be calculated by the following formula [S54]:

$$\eta=mh_{LV}/C_{opt}P_{0}$$

where *m* is the water evaporation rate, $h_{LV}$ is the equivalent evaporation enthalpy of water in aerogels, *C_opt_* refers to the optical concentration on the absorber surface, and *P_0_* is the solar irradiation power of 1.0 sun (1 kW m^-2^). The evaporation rate of the carbon aerogel was 2.25 kg m^-2^ h^-1^.

To obtain the actual vaporization enthalpy, a controlled experiment was conducted [S55]. The bulk water and aerogels with same surface area were simultaneously placed in a dark and closed container together under ambient air pressure and temperature around 25 ℃. The equivalent evaporation enthalpy could be estimated by evaporating water of the samples assuming equal energy input (*U_in_*):

$$U_{in}=h_{0}m_{0}=h_{LV}m_{g}$$

where *h_0_* and *m_0_* are evaporation enthalpy and mass change of bulk water under dark condition, respectively. *m_g_* is mass change of aerogels in the same condition. Therefore, the equivalent evaporation enthalpy ($h_{LV}$) could be estimated according to the equation. For the evaporation enthalpy of bulk water (*h_0_*) was 2440 kJ kg^-1^, the ratio of mass change of bulk water over the mass change of the aerogel was 0.615. The resulted equivalent evaporation enthalpy was 1500 kJ kg^-1^.

For the evaporation enthalpy of bulk water (*h_0_*) was 2440 kJ kg^-1^, the ratio of mass change of bulk water over the mass change of the aerogel was 0.615. The resulted equivalent evaporation enthalpy was 1500 kJ kg^-1^, the average optical concentration in the solar spectrum range was 99.56%, the solar irradiation power under 1.0 sun was 3600 kJ m^-2^. Accordingly, the corresponding energy efficiency (*η*) of the carbon aerogel could be calculated.

**Note S2 Qualitative analysis of the printed aerogels in the heat loss part**

Both the printed composite aerogels can be divided into three regions in the TGA curves.

Stage I (<200 °C): The derived carbon aerogel exhibits significantly lower weight loss (3.3%) compared to the silica-based aerogel (4.7%) in this initial region, indicating reduced moisture/solvent content.

Stage II (Main decomposition): Crucially, the onset temperature of major decomposition shifts dramatically from 322 °C for the silica-based aerogel to 523 °C for the carbon aerogel. Furthermore, the derived carbon aerogel shows remarkably low weight loss at 500 °C (only 4.5% total). Regarding the weight loss in this region, for the silica-based aerogel, it is primarily caused by the pyrolysis of RF in air. In contrast, for the derived carbon aerogel, the weight loss is more concentrated and predominantly occurs above 500 °C. This is mainly attributed to the reaction of amorphous carbon within the derived carbon aerogel with air.

Stage III (700-800 °C): Both the silica-based aerogel and derived carbon aerogel demonstrate high-temperature residue retention, maintaining 65.6% and 64.3% residue at 800 °C, respectively.

This comprehensive quantitative analysis unequivocally confirms the superior thermal stability of the derived carbon aerogel across all temperature regimes, attributed to the formation of a robust carbon skeleton and structurally reinforcing Si-O-C crosslinked networks during pyrolysis.

**Other Supporting Information includes the following:**

Movie S1. 3D patterning process of self-supporting structure

Movie S2. Electrical performance of silica-based nanocomposite aerogels

**Supplementary References**

1. C. Amaral, R. Vicente, V.M. Ferreira, T. Silva, Polyurethane foams with microencapsulated phase change material: Comparative analysis of thermal conductivity characterization approaches. Energ. Buildings. **153**, 392-402 (2017). <https://doi.org/10.1016/j.enbuild.2017.08.019>
2. S. Veiseh, A.A. Yousefi, Compressive behavior and thermal conductivity-density correlation of expanded polystyrene thermal insulators. Iranian Polymer Journal **30**, 849-854 (2021). <https://doi.org/10.1007/s13726-021-00937-6>
3. E.S. Altuntop, D. Erdemir, M.B. Karamis, Experimental investigation of heat conduction characteristics of density-layered stone wool materials. Int. Commun. Heat Mass Transf. **126**, 105334 (2021). <https://doi.org/10.1016/j.icheatmasstransfer.2021.105334>
4. F. Modarresifar, P.A. Bingham, G.A. Jubb, Thermal conductivity of refractory glass fibres a study of materials, standards and test methods. J. Therm. Anal. Calorim. **125**, 35-44 (2016). <https://doi.org/10.1007/s10973-016-5367-0>
5. R. Caps, U. Heinemann, J. Fricke, K. Keller, Thermal conductivity of polyimide foams. Int. J. Heat Mass Tran. **40**, 269-280 (1997). <https://doi.org/10.1016/0017-9310(96)00134-2>
6. W. Cao, X. Cheng, L. Gong, Y. Li, R. Zhang et al., Thermal conductivity of highly porous ceramic foams with different agar concentrations. Mater. Lett. **139**, 66-69 (2015). <https://doi.org/10.1016/j.matlet.2014.08.096>
7. A. Ricklefs, A.M. Thiele, G. Falzone, G. Sant, L. Pilon, Thermal conductivity of cementitious composites containing microencapsulated phase change materials. Int. J. Heat Mass Tran. **104**, 71-82 (2017). <https://doi:10.1016/j.ijheatmasstransfer.2016.08.013>
8. S. Iswar, S. Galmarini, L. Bonanomi, J. Wernery, E. Roumeli et al., Dense and strong, but superinsulating silica aerogel. Acta Mater. **213**, 116959 (2021). <https://doi.org/10.1016/j.actamat.2021.116959>
9. S. Yun, T. Guo, J. Zhang, L. He, Y. Li et al., Facile synthesis of large-sized monolithic methyltrimethoxysilane-based silica aerogel via ambient pressure drying. J. Sol-Gel Sci. Techn. **83**, 53-63 (2017). <https://doi.org/10.1007/s10971-017-4377-0>
10. X. Lu, R. Caps, J. Fricke, C.T. Alviso,R.W. Pekala, Correlation between structure and thermal-conductivity of organic aerogels. J. Non-Cryst. Solids **188**, 226-234 (1995). <https://doi.org/10.1016/0022-3093(95)00191-3>
11. J. Feng, X. Wang, Y. Jiang, D. Du, J. Feng. Study on thermal conductivities of aromatic polyimide aerogels. ACS Appl. Mater. Inter. **8**, 12992-12996 (2016). <https://doi.org/10.1021/acsami.6b02183>
12. S. Wu, A. Du, S. Huang,G. Zu, Y. Xiang at al., Effects of monomer rigidity on the microstructures and properties of polyimide aerogels cross-linked with low cost aminosilane. RSC Adv. **6**, 22868-22877 (2016). <https://doi.org/10.1039/c5ra28152k>
13. Z. Zhu, G. Snellings, M.M. Koebel, W.J. Malfait, Superinsulating polyisocyanate based aerogels: A targeted search for the optimum solvent system. ACS Appl. Mater. Inter. **9**, 18222-18230 (2017). <https://doi.org/10.1021/acsami.7b03344>
14. M.H. Adegun, K.Y. Chan, J. Yang, H. Venkatesan, E. Kim et al., Anisotropic thermally superinsulating boron nitride composite aerogel for building thermal management. Compos. Part A-Appl. S. **169**, 107522 (2023). <https://doi.org/10.1016/j.compositesa.2023.107522>
15. Z. An, R. Zhang, D. Fang, Synthesis of monolithic sic aerogels with high mechanical strength and low thermal conductivity. Ceram. Int. **45**, 11368-11374 (2019). <https://doi.org/10.1016/j.ceramint.2019.02.216>
16. Y. Kong, J. Zhang, Z. Zhao, X. Jiang, X. Shen, Monolithic silicon nitride-based aerogels with large specific surface area and low thermal conductivity. Ceram. Int. **45**, 16331-16337 (2019). <https://doi.org/10.1016/j.ceramint.2019.05.160>
17. Y. Yuan, L. Liu, M. Yang, T. Zhang, F Xu et al., Lightweight, thermally insulating and stiff carbon honeycomb-induced graphene composite foams with a horizontal laminated structure for electromagnetic interference shielding. Carbon. **123**, 223-232 (2017). <https://doi.org/10.1016/j.carbon.2017.07.060>
18. L. Berglund, T. Nissila, D. Sivaraman, S. Komulainen, V.V. Telkki at al., Seaweed-derived alginate-cellulose nanofiber aerogel for insulation applications. ACS Appl. Mater. Inter. **13**, 34899-34909 (2021). <https://doi.org/10.1021/acsami.1c07954>
19. H. Jin, X. Zhou, T. Xu, C. Dai, Y. Gu et al., Ultralight and hydrophobic palygorskite-based aerogels with prominent thermal insulation and flame retardancy. ACS Appl. Mater. Inter. **12**, 11815-11824 (2020). <https://doi.org/10.1021/acsami.9b20923>
20. S. Zhang, X. Huang X, J. Feng, F. Qi, D. E et al., Thermal conductivities of cellulose diacetate based aerogels. Cellulose **27**, 4555-4564 (2020). <https://doi.org/10.1007/s10570-020-03084-y>
21. C.J. Saelices, B. Seantier, B. Cathala, Y. Grohens, Spray freeze-dried nanofibrillated cellulose aerogels with thermal superinsulating properties. Carbohyd. Polym. **157**, 105-113 (2017). <https://doi.org/10.1016/j.carbpol.2016.09.068>
22. N.G. Alburquerque, S. Zhao, N. Adilien N, M.M. Koebel, M. Lattuada et al., Strong, machinable, and insulating chitosan-urea aerogels: Toward ambient pressure drying of biopolymer aerogel monoliths. ACS Appl. Mater. Inter. **12**, 22037-22049 (2020). <https://doi.org/10.1021/acsami.0c03047>
23. S. Zhao, G. Siqueira, S. Drdova, D. Norris, C. Ubert et al., Additive manufacturing of silica aerogels. Nature **584**, 387 (2020). <https://doi.org/10.1038/s41586-020-2594-0>
24. T. Wu, M. Ganobjak, G. Siqueira, Z. Zeng, M. Li et al., 3D printed polyimide nanocomposite aerogels for electromagnetic interference shielding and thermal management. Adv. Mater. Technol. **8**, 2202155 (2023). <https://doi.org/10.1002/admt.202202155>
25. D. Sivaraman, Y. Nagel, G. Siqueira, P. Chansoria, J. Avaro et al., Additive manufacturing of nanocellulose aerogels with structure-oriented thermal, mechanical, and biological properties. Adv. Sci. **11**, 2307921 (2024). <https://doi.org/10.1002/advs.202307921>
26. H. Maleki, S. Montes, N.H. Roodbari, F. Putz, N, Huesing, Compressible, thermally insulating, and fire retardant aerogels through self-assembling silk fibroin biopolymers inside a silica structure-an approach towards 3D printing of aerogels. ACS Appl. Mater. Inter. **10**, 22718-22730 (2018). <https://doi.org/10.1021/acsami.8b05856>
27. E.S. Farrell, N. Ganonyan, I. Cooperstein, M.Y. Moshkovitz, Y. Amouyal et al., 3D-printing of ceramic aerogels by spatial photopolymerization. Applied Materials Today **24**, 101083 (2021). <https://doi.org/10.1016/j.apmt.2021.101083>
28. L. Wang, J. Feng, Y. Luo, Y. Jiang, G. Zhang et al., Versatile thermal-solidifying direct-write assembly towards heat-resistant 3D-printed ceramic aerogels for thermal insulation. Small Methods **6**, 2200045 (2022). <https://doi.org/10.1002/smtd.202200045>
29. K. Wang, Z. Cheng, P. Li, Y. Zheng, Z. Liu et al., Three-dimensional self-floating foam composite impregnated with porous carbon and polyaniline for solar steam generation. J. Colloid Interf. Sci. **581**, 504-513 (2021). <https://doi.org/10.1016/j.jcis.2020.07.136>
30. X. Zhang, L. Ren, J. Xu, B. Shang, X. Liu et al., Magnetically driven tunable 3D structured Fe_3_O_4_ vertical array for high-performance solar steam generation. Small **18**, 2105198 (2022). <https://doi.org/10.1002/smll.202105198>
31. C. Wang, J. Wang, Z. Li, K. Xu, T. Lei et al., Superhydrophilic porous carbon foam as a self-desalting monolithic solar steam generation device with high energy efficiency. J. Mater. Chem. A **8**, 9528-9535 (2020). <https://doi.org/10.1039/d0ta01439g>
32. J. Xiao, Y. Guo, W. Luo, D. Wang, S. Zhong et al., A scalable, cost-effective and salt-rejecting MoS_2_/SA@melamine foam for continuous solar steam generation. Nano Energy **87**, 106213 (2021). <https://doi.org/10.1016/j.nanoen.2021.106213>
33. T. Gao, Y. Li, C. Chen, Z. Yang, Y. Kuang et al., Architecting a floatable, durable, and scalable steam generator: Hydrophobic/hydrophilic bifunctional structure for solar evaporation enhancement. Small Methods **3**, 1800176 (2019). <https://doi.org/10.1002/smtd.201800176>
34. Q. Li, X. Zhao, L. Li, T. Hu, Y. Yang et al., Facile preparation of polydimethylsiloxane/carbon nanotubes modified melamine solar evaporators for efficient steam generation and desalination. J. Colloid Interf. Sci. **584**, 602-609 (2021). <https://doi.org/10.1016/j.jcis.2020.10.002>
35. Y. Guo, F. Zhao, X. Zhou, Z. Chen, G. Yu, Tailoring nanoscale surface topography of hydrogel for efficient solar vapor generation. Nano Lett. **19**, 2530-2536 (2019). <https://doi.org/10.1021/acs.nanolett.9b00252>
36. Y. Sun, J. Gao, Y. Liu, H. Kang, M. Xie et al., Copper sulfide-macroporous polyacrylamide hydrogel for solar steam generation. Chem. Eng. Sci. **207**, 516-526 (2019). <https://doi.org/10.1016/j.ces.2019.06.044>
37. M. Tan, J. Wang, W. Song, J. Fang J, X. Zhang, Self-floating hybrid hydrogels assembled with conducting polymer hollow spheres and silica aerogel microparticles for solar steam generation. J. Mater. Chem. A **7**, 1244-1251 (2019). <https://doi.org/10.1039/c8ta10057h>
38. C. Xiao, W. Liang, Q.M. Hasi, L. Chen, J. He et al., Ag/polypyrrole co-modified poly(ionic liquid)s hydrogels as efficient solar generators for desalination. Materials Today Energy **16**, 100417 (2020). <https://doi.org/10.1016/j.mtener.2020.100417>
39. M.S. Irshad, X. Wang, M.S. Abbasi, N. Arshad, Z. Chen et al., Semiconductive, flexible MnO_2_ NWs/chitosan hydrogels for efficient solar steam generation. ACS Sustain. Chem. Eng. **9**, 3887-3900 (2021). <https://doi.org/10.1021/acssuschemeng.0c08981>
40. J. Yuan, X. Lei, C. Yi, H. Jiang, F. Liu et al., 3D-printed hierarchical porous cellulose/alginate/carbon black hydrogel for high-efficiency solar steam generation. Chem. Eng. J. **430**, 132765 (2022). <https://doi.org/10.1016/j.cej.2021.132765>
41. Z. Sun, Z. Li, W. Li, F. Bian, Mesoporous cellulose/TiO_2_/SiO_2_/Tin-based nanocomposite hydrogels for efficient solar steam evaporation: Low thermal conductivity and high light-heat conversion. Cellulose **27**, 481-491 (2020). <https://doi.org/10.1007/s10570-019-02823-0>
42. H. Wang, A. Du, X. Ji, C. Zhang, B. Zhou et al., Enhanced photothermal conversion by hot-electron effect in ultrablack carbon aerogel for solar steam generation. ACS Appl. Mater. Inter. **11**, 42057-42065 (2019). <https://doi.org/10.1021/acsami.9b12918>
43. X. Deng, Q. Nie, Y. Wu, H. Fang, P. Zhang et al., Nitrogen-doped unusually superwetting, thermally insulating, and elastic graphene aerogel for efficient solar steam generation. ACS Appl. Mater. Inter. **12**, 26200-26212 (2020). <https://doi.org/10.1021/acsami.0c05666>
44. M. He, M.K. Alam, H. Liu, M. Zheng, J. Zhao et al., Textile waste derived cellulose based composite aerogel for efficient solar steam generation. Compos. Commun. **28**, 100936 (2021). <https://doi.org/10.1016/j.coco.2021.100936>
45. L. Li, T. Hu, A. Li, J. Zhang, Electrically conductive carbon aerogels with high salt-resistance for efficient solar-driven interfacial evaporation. ACS Appl. Mater. Inter. **12**, 32143-32153 (2020). <https://doi.org/10.1021/acsami.0c06836>
46. T. Mei, J. Chen, Q. Zhao, D. Wang, Nanofibrous aerogels with vertically aligned microchannels for efficient solar steam generation. ACS Appl. Mater. Inter. **12**, 42686-42695 (2020). <https://doi.org/10.1021/acsami.0c09518>
47. S. Yan, H. Song, Y. Li, J. Yang, X. Jia et al., Integrated reduced graphene oxide/polypyrrole hybrid aerogels for simultaneous photocatalytic decontamination and water evaporation. Appl. Catal. B-Environ. **301**, 120820 (2022). <https://doi.org/10.1016/j.apcatb.2021.120820>
48. W. Xu, Y. Xing, J. Liu, H. Wu, Y. Cuo et al. Efficient water transport and solar steam generation via radially, hierarchically structured aerogels. ACS Nano **13**, 7930-7938 (2019). <https://doi.org/10.1021/acsnano.9b02331>
49. Z. Liu, F. Wu, T. Lv, Y. Qu, Z. Zhang et al., Ti_3_C_2_T_x_/carbon aerogels derived from winter melon for high-efficiency photothermal conversion. Desalination **573**, 117207 (2024). <https://doi.org/10.1016/j.desal.2023.117207>
50. X. Han, S. Ding, L. Fan, Y. Zhou, S. Wang, Janus biocomposite aerogels constituted of cellulose nanofibrils and mxenes for application as single-module solar-driven interfacial evaporators. J. Mater. Chem. A. **9**, 18614-18622 (2021). <https://doi.org/10.1039/d1ta04991g>
51. D.P. Storer, J.L. Phelps, X. Wu, G. Owens,N.I. Khan et al., Graphene and rice-straw-fiber-based 3D photothermal aerogels for highly efficient solar evaporation. ACS Appl. Mater. Inter. **12**, 15279-15287 (2020). <https://doi.org/10.1021/acsami.0c01707>
52. G. Li, Q. Wang, J. Wang, J. Ye, W. Zhou, et al., Carbon-supported nano tungsten bronze aerogels with synergistically enhanced photothermal conversion performance: Fabrication and application in solar evaporation. Carbon 195, 263-271 (2022). <https://doi.org/10.1016/j.carbon.2022.04.023>
53. H. Duan, M. Wang, Z. Zhang, J. Zhen, W. Lv, Biomass-derived photothermal carbon aerogel for efficient solar-driven seawater desalination. J. Environ. Chem. Eng. **11**, 109295 (2023). <https://doi.org/10.1016/j.jece.2023.109295>
54. H. Ghasemi, G. Ni, A.M. Marconnet, J. Loomis, S. Yerci, N. Miljkovic, G. Chen G. Solar steam generation by heat localization. Nat. Commun. **5**, 4449 (2014). <https://doi.org/10.1038/ncomms5449>
55. Y. Guo, F. Zhao, X. Zhou, Z. Chen, G. Yu. Tailoring nanoscale surface topography of hydrogel for efficient solar vapor generation. Nano Lett. **19**, 2530-2536 (2019). <https://doi.org/10.1021/acs.nanolett.9b00252>
